# High reelin expression may explain why a subgroup of entorhinal cortex neurons functions as an initial nucleation site of Alzheimer’s disease

**DOI:** 10.1371/journal.pcbi.1014532

**Published:** 2026-08-25

**Authors:** Asgeir Kobro-Flatmoen, Jagir R. Hussan, Peter J. Hunter, Stig W. Omholt

**Affiliations:** 1 Kavli Institute for Systems Neuroscience, Norwegian University of Science and Technology (NTNU), Trondheim, Norway; 2 Norwegian Health Association Centre for Dementia Research, Norwegian University of Science and Technology (NTNU), Trondheim, Norway; 3 Auckland Bioengineering Institute, The University of Auckland, Auckland, New Zealand; University of Edinburgh, UNITED KINGDOM OF GREAT BRITAIN AND NORTHERN IRELAND

## Abstract

The entorhinal cortex (EC) plays a crucial role in memory functions. Long before the clinical symptoms of Alzheimer’s disease (AD) emerge, it has already undergone significant degeneration, making it a primary site for the onset of the disease. The reasons for this remain elusive. Layer II (LII) neurons of the anterolateral EC are especially prone to display a very early increase in intracellular amounts of amyloid-β peptide (Aβ) and hyperphosphorylated tau protein (p-tau). The expression of the large glycoprotein reelin is extraordinarily high in ECLII neurons compared to most other cortical neurons and proximity ligation assay data and other immunohistochemical data strongly support the notion that reelin binds to Aβ in these neurons. Here, based on the premise that reelin may function as a sink for intracellular Aβ, we show by computational modeling that, in a senescent physiology predisposing to frequent inflammation-driven Aβ42 production bursts over a decades-long period, the intracellular amount of Aβ42-reelin complexes can accumulate to extraordinarily high levels in anterolaterally positioned LII neurons compared to the vast majority of cortical neurons. This is consistent with experimental data showing that intracellular accumulations of Aβ42 positive material ranged from 20 to 80% of total soma volume in EC neurons from patients with idiopathic AD. Based on known tau protein biology, we also show that this extreme intracellular aggregation that overloads the lysosomal degradation machinery, manifesting chronic homeostatic dysregulation, can lead to the production of p-tau fragments prone to aggregation. Together, our findings may contribute to the resolution of why the EC is so strongly associated with the very early etiology of AD.

## Author summary

Why the entorhinal cortex, located adjacent to the hippocampus in the medial temporal lobe, is a major cradle of the onset of Alzheimer’s disease still eludes us. In contrast to most other cortical neurons, a subgroup of entorhinal cortex neurons express very high levels of the large glycoprotein reelin. In complete agreement with experimental data, we show by computational modeling that in the elderly with a senescent physiology predisposing to frequent inflammation events over a decades-long period, a complex formed by reelin and amyloid-β can accumulate in these neurons to fill more than 80% of the cell body. Due to the detrimental cascading chain of events that is likely to accompany this chronic homeostatic dysregulation, the intraneuronal expression level of reelin appears to qualify as an important factor for the onset of Alzheimer’s disease.

## Introduction

Long before the appearance of clinical symptoms in the course of Alzheimer´s disease (AD), increased intracellular amounts of amyloid-β peptide (Aβ) followed by hyperphosphorylated tau protein (p-tau) are predominantly restricted to neurons of layer II (LII) of the entorhinal cortex (EC), especially those of the anterolateral domains of EC [1,2]. Multiple studies show that these neurons are typically among the first to degenerate in the course of the disease [3–6]. Since ECLII neurons are integral to the generation of spatial and relational representations on which our memory system depends, it helps explain why the initial symptoms of most, if not all types of AD include impaired memory.

More than 20 years ago, it was convincingly shown by D’Andrea et al. [7,8] that (i) Aβ42 accumulated selectively as discrete granules associated with lysosomes and/or their derivatives in the soma of principal neurons in the EC of AD brains (72–78 years), (ii) the volume of Aβ42 positive material ranged from 20-80% of total soma volume, and (iii) regions that were abundantly populated with principal neurons displaying excessive Aβ42 accumulations also contained evidence of neuronal lysis. To the best of our knowledge, these findings have not been experimentally refuted. Instead, a solid amount of data accumulated in the past 20 years shows that lysosomal dysfunction is a commonly observed abnormality in proteinopathic neurodegenerative diseases [9]. And recent findings in mice [10] suggest that there is in fact a causal link between a dysfunctional endosome-autophagosome-lysosome pathway (EALP), pronounced intraneuronal accumulation of Aβ, and loss of neuronal structural integrity leading to cell death and subsequent plaque formation.

The diameter of the 4.5 kDA Aβ42 molecule is ≈2 nm [11,12]. If we, based on the available data [13], take the soma volume of an anterolaterally positioned LII neuron to be ≈5,000 $\mu m^{3}$, and conservatively assume that Aβ42 peptides are packed 1/5 of what can be obtained by random close packing of spheres [14], allowing rather sparse packing of monomeric and oligomeric Aβ42 [15,16], it follows that the copy number of Aβ42 must be ≈1x10^11^ to occupy 80% of the soma volume. Suppose that this number has accumulated over 10 years and that there has been no decay, the mean production rate must have been ≈1x10^6^ molecules per hour, which is in the upper range of what has been observed in *E. coli* grown in reach medium [17]. Taking into account that we have assumed no Aβ42 decay, a very sparse packing, and that the estimated maximum Aβ42 production rate in an anterolaterally positioned ECLII neuron is estimated to be ≈1.1x10^5^ per hour [13], this figure appears unrealistically high. This strongly suggests that to reach the observed 80% soma volume occupancy of Aβ42 positive granules in EC cells [7], Aβ42 must have accumulated together with one or more much larger molecules.

The function of the large glycoprotein reelin in adult mammalian brains includes synaptic modulation, induction of enhanced spine density, and promotion of long-term potentiation [18]. The most anterolaterally positioned LII neurons are unique among cortical excitatory neurons by expressing extraordinarily high levels of reelin (dubbed Re^+^ECLII neurons in the following). In these neurons, Aβ co-localizes with reelin immunohistochemically [19], and it was recently shown that reelin and Aβ42 most likely make a stable complex (A$\beta 42_{reelin}$) [20]. Since the volume of the 388 kDa reelin molecule is ≈86 times greater than that of an Aβ42 molecule, this provides a possible mechanism by which excessive amounts of Aβ42 positive granules can accumulate in Re^+^ECLII neurons if they are exposed to frequent bursts of Aβ42 production induced, for example, by inflammation [21–23].

Here, using a computational model based on and integrating a wide range of experimental data, we show that in the context of a senescent physiology predisposing to frequent inflammation events that trigger Aβ42 production [24,25], the amount of A$\beta 42_{reelin}$ in Re^+^ECLII neurons can accumulate to occupy more than 50% of the soma volume over a period of about two decades, while there is no marked accumulation of A$\beta 42_{reelin}$ in cortical neurons expressing low levels of reelin (LR neurons). Furthermore, on the basis of known tau protein biology, we demonstrate that it is fully conceivable that this accumulation is causally related to the observation that high intracellular levels of aggregation-prone p-tau fragments appear much earlier in Re^+^ECLII neurons than in other cortical neurons. Intriguingly, simply by reducing the affinity between reelin and Aβ42, the model recapitulates the phenotype of a rare mutation in the gene coding for reelin, recently shown to cause extreme resistance to AD [26].

By providing a mechanistic rationale for the extremely high intracellular accumulation of A$\beta 42_{reelin}$ in Re^+^ECLII neurons, our results support the opinion that Aβ accretion can lead to lysis of neurons [7,10,27]. Based on their finding that there is a direct association between selective intraneuronal accumulation of Aβ and neuronal loss in AD, Caramello *et al*. [28] recently raised the question - posed by several others over the past three decades - of whether the mechanisms responsible for the accumulation of intraneuronal Aβ could explain the fundamental link between Aβ and p-tau in the genesis and progression of AD. Our results provide an affirmative answer to this question.

## Computational model

### Outline of the model

The basic component of the computational model is a differential equation model of the relevant dynamics in a single cortical neuron. The model describes the per hour rate of change of the intracellular copy numbers of free Aβ42 ([Aβ]), free reelin ([*Reelin*]), A$\beta 42_{reelin}$ tagged for degradation by the EALP ([$A\beta_{reelin,L}$]), Aβ42 similarly tagged for degradation by the EALP ([$A\beta_{L})$]), phosphorylated GSK3β prevented from inducing tau hyperphosphorylation ([$GSK3\beta_{p}$]), free hyperphosphorylated tau ([$tau_{p}$]), and aggregation-prone p-tau fragments associated with the EALP ([$tau_{p,agg}$]).

The model is designed to capture the effect of increasing the production rate of Aβ42 from a normal baseline level to a markedly higher level for a given number of hours before allowing the production rate to return to the baseline level. This mimics the effect of the onset and end of a short-term infectious or sterile inflammation event [22,29].

To predict the long-term time evolution of the seven state variables over [$\Omega_{1},\Omega_{2}$] years caused by recurrent EC inflammation events of varying durations and of multifactorial peripheral or central nervous system origins that characterize senescent physiology [21,22,30–32], we run the dynamic model according to the information provided by an inflammation event vector *I*. This vector was created by a stochastic process that, guided by published statistics on the prevalence of inflammatory events (S1 Appendix), defined when an inflammation event occurred and how long it lasted. For simplicity, we let the probability of an inflammation event in a given month in [$\Omega_{1},\Omega_{2}$] increase linearly from 0 to Ψ, and let its duration be uniformly sampled from the set {$D_{min},..,D_{max}$} days (see S1 Fig for further details).

During an inflammation period, the production rate of [Aβ] was increased to a maximum constant value ($\alpha_{infection}$), otherwise it was kept at a constant baseline value ($\alpha_{baseline}$). Unfortunately, quantitative data on the variability of the duration of brain inflammation events as a function of age that could guide the parameterization underlying *I* do not appear to be available. However, based on data from the literature and statistics available on the Web site of the US Government Centers for Disease Control and Prevention, we can conservatively estimate that, on average, a person will be exposed to around 70–75 brain inflammation events from 50 to 72 years (see S1 Appendix for details). This estimate was used to parameterize Ψ. All other parameter values of the dynamic model were kept constant throughout the 22-year period.

The computational model was coded in Python. The SciPy Python library [33] was used to generate the time evolution of the differential equation system in the period [$\Omega_{1},\Omega_{2}$] (scipy.integrate.solve_ivp with the Radau solver). The code and data that produce the results and figures in the main text and the Supplementary Information are available on Zenodo (https://zenodo.org/records/15680979).

### The dynamic model

The dynamic model is an extended version of a previously published model [13] that focused on the short-term dynamics of [Aβ], [*Reelin*], [$A\beta_{reelin}$] and [$GSK3\beta_{p}$]. Since available data clearly support that Aβ and p-tau are integral parts of an evolutionary very old intraneuronal immune response to acute microbial or viral infection of the mammalian brain [34–42], the previous model was deliberately designed to capture key features of the dynamics of a single immune response event in native physiology over a time scale of hours [13] and not the long-term cumulative effects of recurrent activation of this immune response. The current model addresses the much more daunting task of projecting the dynamics over a two decade period in the context of a senescent physiology exposed to such recurrent activation through chronic reinvasion and reinfection of pathogens, chronic systemic inflammation, or an interaction between the two. The model is oblivious to what causes a single inflammation event as it only assumes that such an event is capable of triggering an increase in Aβ42 production and that the maximum production rate is higher in Re^+^ECLII neurons than in LR neurons (see reference [13] for a justification).

The dynamic model is given by Eqs. (1)-(7) below. To facilitate understanding, the functions (8)-(11) contained in four of the seven equations are listed separately.


$$\begin{array}{c} \frac{d[A\beta ]}{dt}=\alpha (t)-\beta [A\beta ]-\gamma [A\beta ][Reelin], \end{array}$$ (1)

$$\begin{array}{c} \frac{d[Reelin]}{dt}=\tau -\rho [Reelin]-\gamma [A\beta ][Reelin], \end{array}$$ (2)

$$\begin{array}{c} \frac{d[A\beta_{reelin,L}]}{dt}=\gamma [A\beta ][Reelin]-\delta H_{1}([A\beta ],\theta_{1},n_{1})S_{0}^{T_{1}}([A\beta_{reelin,L}]), \end{array}$$ (3)

$$\begin{array}{cc} \frac{d[A\beta_{L}]}{dt} & =\beta [A\beta ]-\sigma H_{1}([A\beta ],\theta_{1},n_{1})S_{0}^{T_{2}}([A\beta_{L}]), \end{array}$$ (4)

$$\begin{array}{c} \frac{d[GSK3\beta_{p}]}{dt}=\eta [Reelin]\left(1-\frac{\left[GSK3\beta_{p}\right]}{GSK3\beta_{tot}}\right)-\kappa [GSK3\beta_{p}], \end{array}$$ (5)

$$\begin{array}{cc} \frac{d[tau_{p}]}{dt} & =\upsilon \left(1-\frac{\left[GSK3\beta_{p}\right]}{GSK3\beta_{tot}}\right)-\mu [tau_{p}] \end{array}$$ (6)

$$\begin{array}{c} \frac{d[tau_{p,agg}]}{dt}=\phi \mu [tau_{p}]-\omega H_{1}([A\beta ],\theta_{1},n_{1})H_{2}([A\beta_{reelin,L}],\theta_{2},n_{2})[tau_{p,agg}], \end{array}$$ (7)

where


$$\begin{array}{c} H_{1}([A\beta ],\theta_{1},n_{1})=\left(\frac{1}{1+{\left(\frac{\left[A\beta \right]}{\theta_{1}}\right)}^{n_{1}}}\right), \end{array}$$ (8)

$$\begin{array}{c} H_{2}([A\beta_{reelin,L}],\theta_{2},n_{2})=\left(\frac{1}{1+{\left(\frac{\left[A\beta_{reelin,L}\right]}{\theta_{2}}\right)}^{n_{2}}}\right), \end{array}$$ (9)

$$\begin{array}{c} S_{0}^{T_{1}}([A\beta_{reelin,L}])=\frac{1}{2}(T_{1}+[A\beta_{reelin,L}]-|[A\beta_{reelin,L}]-T_{1}|), \end{array}$$ (10)

$$\begin{array}{c} S_{0}^{T_{2}}([A\beta_{L}])=\frac{1}{2}(T_{2}+[A\beta_{L}]-|[A\beta_{L}]-T_{2}|), \end{array}$$ (11)

The premises underlying the above model are:

### Equation (1)

The production rate of Aβ42 (α(t)) is at a very low constitutive baseline level ($\alpha_{baseline}$) or at a much higher constitutive level when the cell is exposed to an inflammation event ($\alpha_{infection}$). The degradation of Aβ42 is a first-order process (β[Aβ]), and there is a second-order binding reaction between Aβ42 and reelin (γ[Aβ][*Reelin*]). We assume a 1:1 binding of the reelin to Aβ42 (see Discussion on the effect of a different stoichiometry).

### Equation (2)

Reelin production is constitutive (τ), a first-order process (ρ[Reelin]) labels free reelin for degradation, and the second-order binding reaction between Aβ42 and reelin is identical to the last term in Eq. (1). Free reelin labeled for degradation might, under certain conditions, accumulate intracellularly (see the descriptions of Eqs. (3) and (4) below). However, since this is unlikely to be functionally significant in an AD context, we have not considered this possibility.

### Equation (3)

The subscript L in [$A\beta_{reelin,L}$] indicates that the A$\beta 42_{reelin}$ complex is rapidly tagged for degradation by the EALP. In native physiology, it is reasonable to assume that the degradation can be approximated by first order kinetics [13]. However, in senescent physiology, this is unlikely to be the case, since the disruption of the EALP consistently appears as an early and progressive characteristic of the pathophysiology of AD [43], leading to accumulation of degradation-tagged cellular cargo. A solid body of data implicates that dysfunction of the lysosomal vacuolar (H+)–adenosine triphosphatase (v-ATPase) proton pump is of particular significance [44]. Both Aβ and β-C-terminal fragment (β-CTF), another cleavage product of the amyloid precursor protein (APP), appear to be capable of altering v-ATPase to cause lysosomal alkalinization leading to a general decrease in lysosomal degradation [9,10,45]. Since the expression level β-CTF is likely to be highly correlated with the expression level of APP, which is closely related to the expression level of its proteolytic metabolite Aβ [46,47], we used only [Aβ] in the inverse Hill function (8) to express that when the levels of Aβ42 and β-CTF pass a critical threshold, the processing capacity of the EALP starts to decline rapidly (see S2 Fig).

The function (10) expresses the assumption that the degradation of [$A\beta 42_{reelin,L}$] is a first order process $\delta [A\beta_{reelin,L}]$ up to a given copy number threshold *T*_1_. When [$A\beta_{reelin,L}$] reaches *T*_1_, the processing machinery becomes saturated, so the number degraded per hour becomes constant ($\delta T_{1}$) (S3 Fig). This is likely to be a conservative assumption because as the number of EALP bodies associated with accumulation increases, this may lead to a demand for v-ATPase that is beyond what its production machinery has evolved to handle, causing alkalinization and therefore an even stronger decline in degradation capacity. Note that the equation assumes that the saturation kinetics also applies when there is no Aβ/β-CTF driven reduction in the processing capacity of the EALP. Smooth versions of the functions (10) and (11) [48] were used in the numerical simulations as required by the numerical solver used (see the caption of S3 Fig for further details).

### Equation (4)

The processing rate of intracellular Aβ42 tagged for degradation in the EALP ([$A\beta_{L}$]) [49–52] follows the same kinetics as that of A$\beta 42_{reelin}$ tagged for degradation (see S3 Fig).

### Equation (5)

By binding to its main receptor in the brain, the apolipoprotein E receptor 2 (ApoER2), reelin triggers a signaling cascade that strongly inhibits the otherwise constitutively active glycogen synthase kinase 3β (GSK3β) [53–55], which is one of the main kinases that phosphorylates tau. A major reason for our focus on this kinase is that the expression of active GSK3β is strongly correlated with the tangle-like inclusions that are initially observed in ECLII neurons [56] and that interaction with Aβ42 reduces the ability of reelin to inhibit GSK3β kinase activity [57]. The equation expresses the assumption that the phosphorylation rate of the serine 9 (S9) epitope on GSK3β, whose phosphorylation causes its inhibition [58], is a function of the reelin level and the total copy number of S9-unphosphorylated GSK3β ($GSK3\beta_{tot}$) (η is a constant) and that the dephosphorylation rate is a first order process (κ[$GSK3\beta_{p}$]). See reference [13] for further justification of the phosphorylation term. This equation is based on the assumption that reelin is present in sufficient quantity to influence the GSK3β pathway also in the vast majority of LR cortical neurons. This is supported by a quantitative analysis of brain image data collected from 3, 12 and 18-month-old wild type rats, which showed that almost all of the ≈172,500 neurons analyzed, uniformly sampled from each layer in five different cortical regions, possessed a reelin signal clearly above the background signal [13]. We anticipate that this result translates to humans.

### Equation (6)

The rate of p-tau production is proportional to the fraction of S9-unphosphorylated GSK3β and υ is the maximum production rate. The rate at which p-tau is marked for degradation in the EALP [59] is a first order process (μ[$tau_{p}$]). In a normal mature neuron, the tau concentration is estimated to be ≈2 μM and practically all tau is assumed to be bound to microtubules [60]. The protein is recovered in three main states (soluble, oligomeric, and fibrillized) from the brains of AD subjects, and although there is as much normal cytosolic tau in the AD brain as in the normal aged brain, the level of abnormally hyperphosphorylated tau is several times higher and up to 40% of the tau is oligomeric [60]. This suggests that the intracellular level of normal tau is under homeostatic feedback control and that p-tau is not part of this regulation, which supports the use of the simple production term in Eq. (6).

### Equation (7)

The EALP appears to contribute both to the production of proaggregating forms by fragmentation of tau and to the clearance of tau aggregates [61]. The phosphorylation status of tau is probably crucial for the former process, since hyperphosphorylation has been shown to induce a more extended tau conformation that exposes the aggregation-prone repeat domains (R1–R4) [62,63], thereby promoting the formation of p-tau oligomers. The removal of such oligomers involves the EALP [64,65], and the interaction between p-tau and the EALP appears to be crucial with respect to the further aggregation of p-tau. Specifically, truncated tau segments formed by proteolytic activity seem to be important in this process. This is because such segments are delivered to lysosomes where, rather than being translocated into the lumen, they tend to get stalled at the membrane and cleaved by cathepsin L into smaller fragments to the point where only the R1-R4 region remains. Crucially, these smaller R1-R4 containing truncated tau fragments are much more aggregation-prone than full length tau [61,66], and impairment of the EALP is known to result in the formation of aggregated p-tau [65]. Based on this, the production term in Eq. (7) says that a fraction ϕ≤1 of $tau_{p}$ tagged for EALP degradation is on the surface of lysosomes converted to truncated forms of p-tau that are prone to aggregation [$tau_{p,agg}$]. Since tau fragments are generated via chaperone-mediated autophagy, they can be produced even if lysosomal fusion is blocked by lysosomal alkalinization [67] inasmuch as lysosomal proteases remain operative [68]. This suggests that the production term in Eq. (7) also applies during Aβ/β-CTF driven lysosomal alkalinization.

The function *H*_2_(.) (9) in the degradation term expresses the assumption that there is an inverse sigmoidal relationship between [$A\beta_{reelin,L}$] and the rate at which the truncated forms of p-tau are degraded. Hence, the lysosomal uptake rate of the truncated p-tau forms will in the non-inflammatory case be according to first order kinetics (ω[$tau_{p,agg}$]) until the number of EALP bodies containing large amounts of A$\beta 42_{reelin}$ starts to predominate (S4 Fig). A biological rationale for this mechanism is that the accumulation of A$\beta 42_{reelin}$ in lysosomes will, above a certain threshold, start to impact the processing rate of tau segments on the lysosomal surface, for example, by pathological alkalinization of lysosomes due to a higher demand for v-ATPase than what its production machinery has evolved to handle. Due to this, the residence time of tau segments on the lysosomal surface will also increase when lysosomes are not alkalinized during Aβ42 production bursts and thus lead to enhanced production of aggregation-prone tau fragments.

We deliberately bounded the model to capture the direct effects of chronic activation of an intraneuronal immune response. Thus, we did not include the positive feedback loop that is likely to arise between [Aβ] and [$tau_{p,agg}$] at some stage. A similar loop between [Aβ] and [$A\beta_{reelin,L}$] is also conceivable. The inclusion of such loops would impact the results, but due to the paucity of data that could guide the parameterizations of when such loops would start to tick in and how strong they would be, we think their inclusion would be premature. The main reason why we think this omission is not a major weakness is that such cascade effects are at least partially covered by the way we have designed the inflammation event vector that drives the induction of enhanced Aβ42 production. The imposed increase in the frequency of inflammation events as a function of age can be viewed as a proxy for a variety of intracellular and extracellular processes that influence Aβ42 production that are not explicitly included in the model, such as the above positive feedback loops.

### Parameterization of the differential equations

The parameter values used are listed in Table 1. The parameter values that differ in LR vs Re^+^ECLII neurons are highlighted by light blue shading. See reference [13] for a justification of the values used for $\alpha_{baseline}$, $\alpha_{infection}$, β, γ, τ, ρ, δ, η, $GSK3\beta_{tot}$, κ and soma volume.

**Table 1:** Parameter values used to describe LR and Re^+^ECLII neurons in the dynamic model.

	LR	Re^+^	Units
$\alpha_{baseline}$	1560	7020	$\text{molecules}\cdot h^{-1}$
$\alpha_{infection}$	4.4x10^4^	1.09x10^5^	$\text{molecules}\cdot h^{-1}$
β	0.1	0.1	*h* ^–1^
γ	0.005	0.005	${\text{molecules}}^{-1}\cdot h^{-1}$
τ	1.45x10^4^	7.9x10^4^	$\text{molecules}\cdot h^{-1}$
ρ	0.05	0.05	*h* ^–1^
δ	0.02	0.02	*h* ^–1^
$\theta_{1}$	2.36x10^5^	2.36x10^5^	*molecules*
*n*_1_	3	3	1
*T*_1_	9.5x10^5^	9.5x10^5^	*molecules*
σ	0.1	0.1	*h* ^–1^
*T*_2_	3.9x10^5^	3.9x10^5^	*molecules*
η	12	12	*h* ^–1^
$GSK3\beta_{tot}$	5x10^4^	2.5x10^5^	*molecules*
κ	0.65	0.65	*h* ^–1^
υ	1.2x10^4^	1.2x10^4^	$\text{molecules}\cdot h^{-1}$
μ	0.1	0.1	*h* ^–1^
ϕ	1	1	1
ω	0.01	0.01	*h* ^–1^
$\theta_{2}$	1x10^8^	1x10^8^	*molecules*
*n*_2_	3	3	1
soma volume	4846	4846	$\mu m^{3}$

We let $\theta_{1}$ be ≈80% of the maximum cellular concentration of free Aβ42 during an inflammation event. The value of *n*_1_ reflects that the effect of the Aβ/β-CTF driven alkalinization of lysosomes is assumed to be a moderate sigmoidal process. The thresholds *T*_1_ and *T*_2_ in functions (10) and (11) were allowed to be ≈50% above the steady state copy number of [$A\beta_{reelin,L}$] and [$A\beta_{L}$] in LR neurons during an inflammation event in native physiology, assuming there is no effect of lysosomal alkalinization. Since there is little evidence for accumulation of Aβ42 and p-tau in EALP under normal conditions in native physiology, we let σ = μ = β. The value of υ reflects the assumption that the neuron can increase its production of p-tau quite quickly when reelin does not inhibit the GSK3β pathway. Due to reduced translocation of R1-R4 containing tau fragments across the lysosomal membrane, we let ω be 1/10 the value of μ. The value of *n*_2_ assumes the existence of a moderate sigmoidal dose-response relationship. The value of $\theta_{2}$ assumes that [$A\beta_{reelin,L}$] has to be about ten times higher than the number experienced by an LR neuron in native physiology exposed to an inflammation event lasting a few days before it begins to influence the degradation rate of [$tau_{p,agg}$].

## Results

### Dynamic response to a short-term inflammation event

The predicted dynamics of six of the seven state variables associated with a single short-term inflammatory event in a LR and a Re^+^ECLII neuron are shown in S5 and S6 Figs. The main distinctions between the two cases are that in the latter, the level of [$A\beta_{reelin,L}$] increases approximately seven times and decreases much slower after the cessation of the inflammation event. The parameter values that are different in the two cases are the [Aβ] production rates $\alpha_{baseline}$ and $\alpha_{infection}$, the constitutive production rate of reelin (τ), and the size of $GSK3\beta_{tot}$. These differences are justified in reference [13].

### Long-term dynamic response to recurrent inflammation

**Fig 1:**
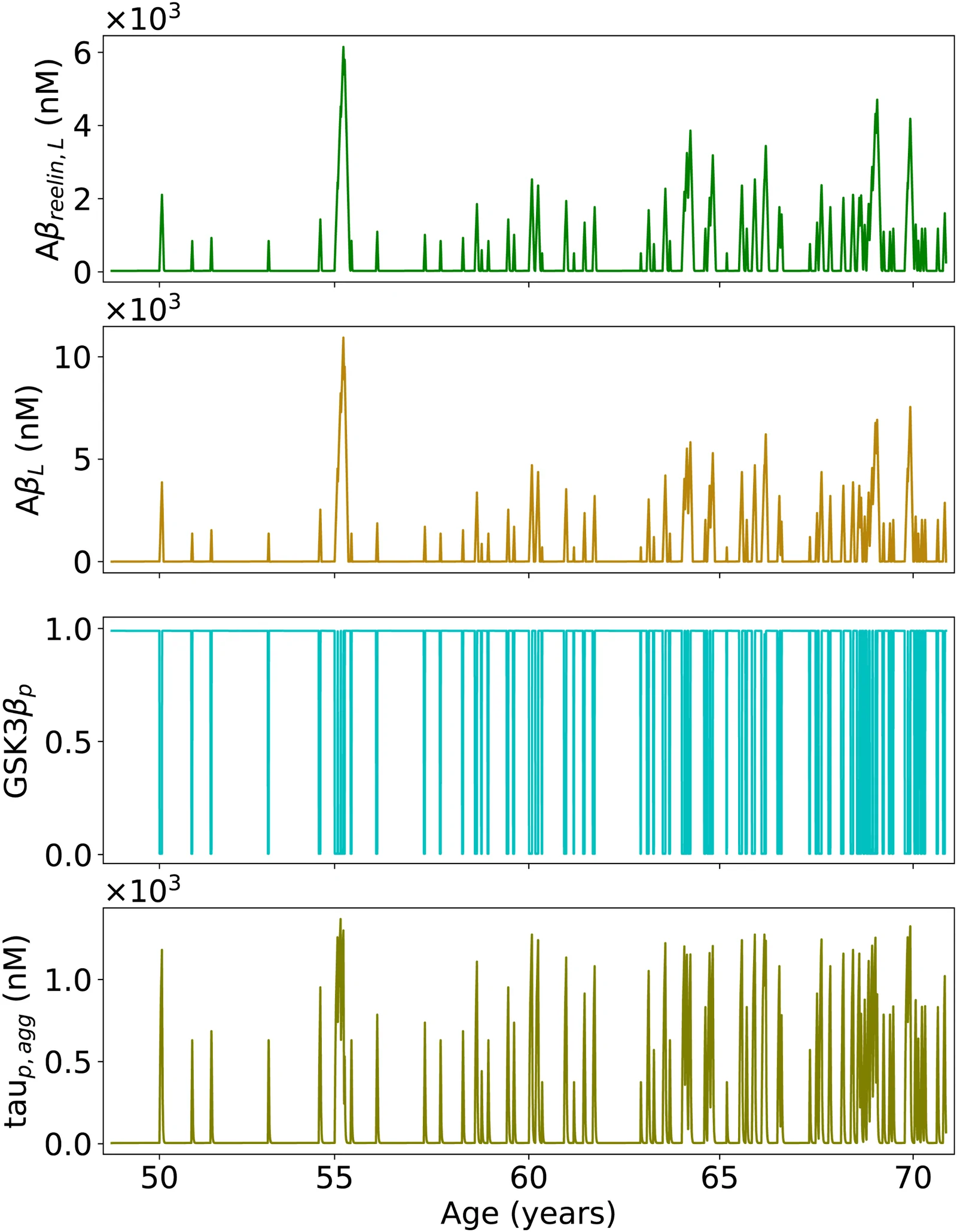
Predicted time evolution of four of the seven state variables in an LR neuron over 22 years. GSK3 $\beta_{p}$ is expressed as proportion of the total number of GSK3 β. The parameter values used to generate the inflammation event vector *I*: $\Omega_{1}$ = 50 years, $\Omega_{2}$ = 72 years, Ψ = 0.6, $D_{min}$ = 5 days, $D_{max}$ = 29 days.

**Fig 2:**
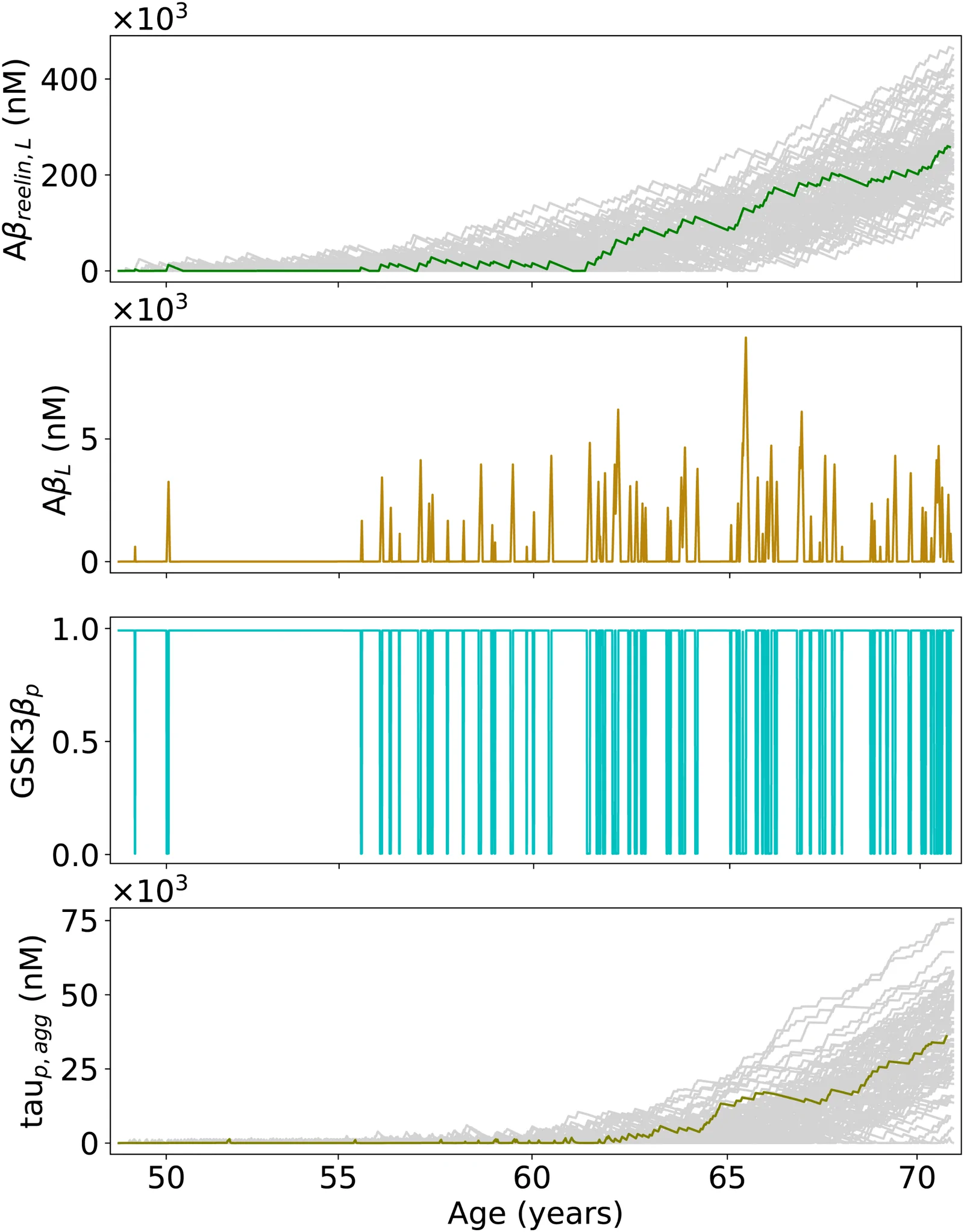
Predicted time evolution of four of the seven state variables in a Re^+^ECLII neuron over 22 years. *I* is identical to the one used for the LR neuron (Fig 1).

By running the full computational model where the Aβ42 production (Eq. (1)) was successively evoked for a given time period according to the vector of inflammation events (*I*) as defined above, we obtained the development of the seven state variables over a 22-year span ($\Omega_{1}$ = 50 years,$\Omega_{2}$ = 72 years) in an LR and a Re^+^ECLII neuron (Figs 1 and 2, only four variables are shown).

In an LR neuron (Fig 1), the model predicts that [$A\beta_{reelin,L}$], [$A\beta_{L}$] and [$tau_{p,agg}$] will increase during each inflammation event, but that their decrease due to degradation between each bout will be large enough to prevent a steady increase in the three state variables. The proportion of GSK3β being phosphorylated at S9 [$GSK3\beta_{p}$] is predicted to be ≈1 between inflammation events and ≈0 during an inflammation event.

In the Re^+^ECLII neuron case (Fig 2), the model predicts that the time series pattern of [$A\beta_{L}$] and [$GSK3\beta_{p}$] will be similar to that of an LR neuron, while [$A\beta_{reelin,L}$] and [$tau_{p,agg}$] will start to accumulate steadily at a given time point. Since *I* is a stochastic vector, we have illustrated the spread in the temporal evolution of [$A\beta_{reelin,L}$] and [$tau_{p,agg}$] obtained from using 100 additional versions of *I* based on the same parameterization as the green and olive lines in panels 1 and 4 (light gray lines). We see that the time development of the two state variables can vary substantially depending on the inflammation history given by *I*, but with the given parameterization of *I*, the model consistently predicts that [$A\beta_{reelin,L}$] and [$tau_{p,agg}$] will accumulate intraneuronally in Re^+^ECLII neurons (Fig 2, panel 1). The volume of a A$\beta 42_{reelin}$ complex is at least 470 *nm*^3^ assuming a density of 1.37 g/*cm*^3^ [12]. Assuming that the complexes are packed about a third as densely as can be obtained by a random close packing of spheres, a [$A\beta_{reelin,L}$] value of 375 μM corresponds to a soma volume occupancy of ≈50%. Thus, the model appears capable of quantitatively recapitulating the experimental data reported by D´Andrea et al. [7,8].

It should be noted that [$A\beta_{reelin,L}$] and [$tau_{p,agg}$] can also increase steadily in an LR neuron if we dramatically increase the number and mean duration of inflammation events. However, the model then predicts that the soma volume occupancy of [$A\beta_{reelin,L}$] in a Re^+^ECLII neuron will be much higher than 100%.

The extreme accumulation of Aβ42 positive material in EC neurons of AD subjects [7] implies that the mean degradation rate (and export) of this material must have been substantially lower than the mean production rate for a long period of time. If we exchange the saturation function $S_{0}^{T_{1}}$(.) with [$A\beta_{reelin,L}$] and keep all remaining parameters fixed, we do not get any accumulation of A$\beta 42_{reelin}$ in the Re^+^ECLII neurons. This supports the notion that degradation must begin to follow zero order kinetics when [$A\beta_{reelin,L}$] reaches a given threshold. This prevents the return to the baseline level in non-inflammatory periods and leads to accumulation of A$\beta 42_{reelin}$, manifesting chronic homeostatic dysregulation.

Although the results must be interpreted with caution due to the paucity of experimental data that could have further constrained the choice of parameter values, they clearly indicate that enhanced constitutive reelin expression in Re^+^ECLII neurons makes these neurons much more vulnerable to the onset of AD than LR neurons. As shown in the following, this contention is supported by recent experimental data.

### The effect of reduced affinity between Aβ42 and reelin

A presumably rare mutation in the reelin gene (*RELN*) has recently been claimed to cause extreme resistance to EC degeneration in an individual with the autosomal dominant familial AD (ADFAD) PSEN1-E280A mutation [26]. The individual remained cognitively intact until age 67, while carriers of PSEN1-E280A usually show clinical AD symptoms in their mid to late 40s. The reelin mutation appears to prevent EC neurons from developing a severe tau tangle burden, despite being exposed to a high Aβ load [26].

**Fig 3:**
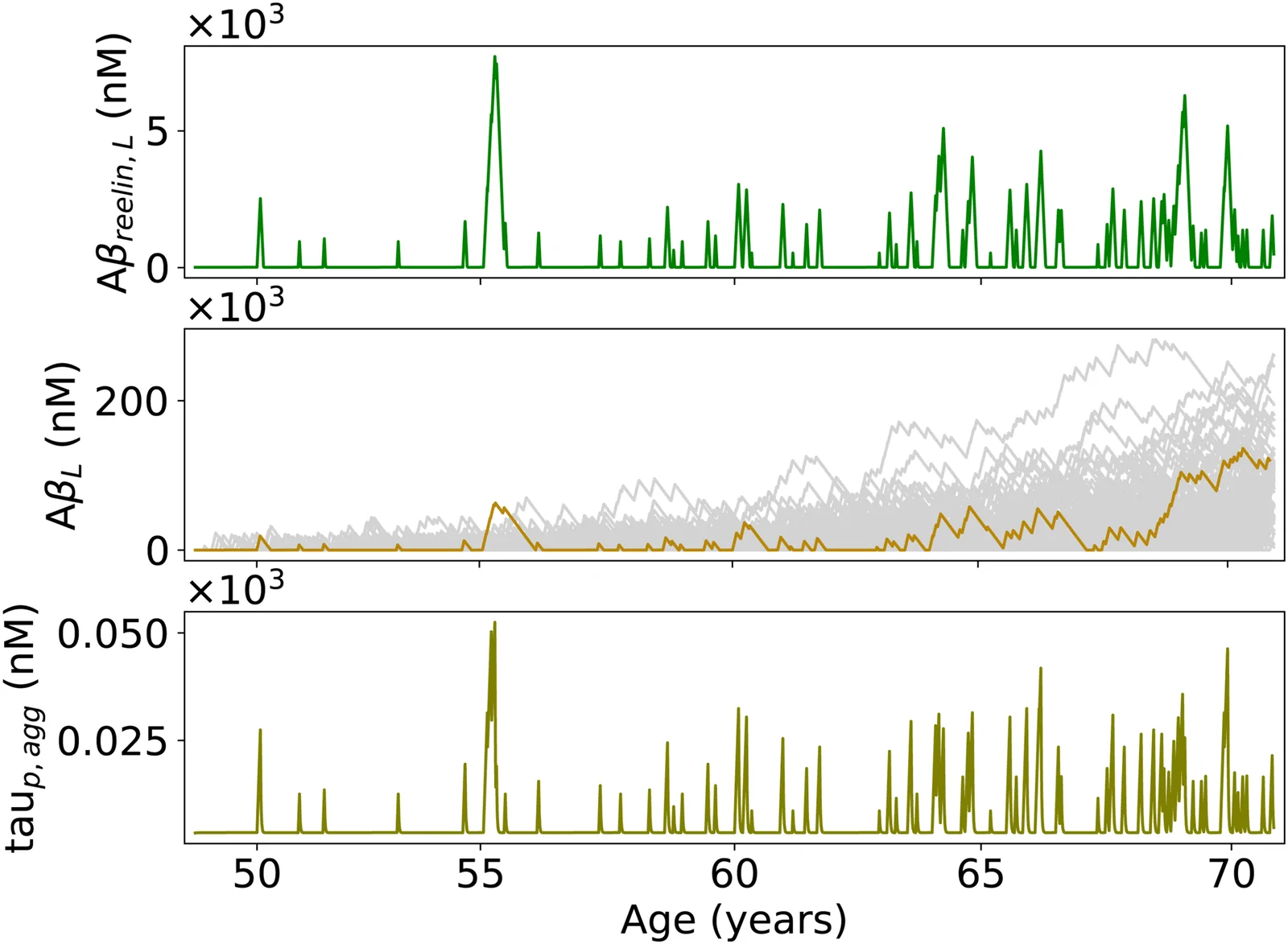
Predicted time evolution of three of the seven state variables over 22 years in a Re^+^ECLII neuron carrying a mutated *RELN* allele. Here, we let $\gamma =1\times 10^{-8}$, while keeping all other parameters fixed. *I* is identical to the one used for the wild type Re^+^ECLII neuron (Fig 2).

One possible explanation is that the mutation (*RELN-COLBOS*) causes a dramatic reduction in the affinity between Aβ42 and reelin. Letting the value of the parameter γ (Eqs. (1)-(3)) become much less in a Re^+^ECLII neuron than that used to produce Fig 2 (assuming the reelin mutation is dominant), while keeping all other parameters fixed and not including the effect of the PSEN1-E280A mutation, the model predicts that there will be marginal accumulation of A$\beta 42_{reelin}$ and aggregation-prone p-tau fragments (Fig 3, panels 1 and 3). In contrast, it predicts a substantial increase in [$A\beta_{L}$] (Fig 3, panel 2), consistent with the high Aβ load observed in the *RELN-COLBOS* case [26]. [$GSK3\beta_{p}$] is predicted to be ≈1, regardless of whether there is an infection event or not. Lopera *et al*. reported that *RELN-COLBOS* significantly increased tyrosine phosphorylation of the intracellular adaptor protein Disabled-1 (Dab1) compared to wild-type (WT) *RELN* in primary culture mouse cortical neurons [26]. Dab1 activation leads to the phosphorylation of GSK3β, which in turn inhibits the formation of p-tau. Thus, since the predictions of the model are clearly in agreement with these primary cortical neuron data and other key phenotypic effects of *RELN-COLBOS*, such as the limited EC tau tangle burden, it seems worthwhile to test to what extent *RELN-COLBOS* affects the affinity between reelin and Aβ.

### Temporal increase in [$A\beta_{reelin,L}$] in layer II vs. layer III neurons

Neuronal death in subjects developing AD probably starts several years earlier in ECLII than in ECLIII [3,4,69]. If this neuronal death is causally related to the soma volume occupancy of A$\beta 42_{reelin}$, the increase in [$A\beta_{reelin,L}$] in ECLIII will have to be much slower than in ECLII. To test whether the model predicts this, we quantified the expression levels of reelin along the lateral-to-medial extent (i.e., close to the rhinal fissure vs increasingly further away) in Re^+^ECLII and Re^+^ECLIII neurons in three 3 month and three 12 month old wild-type rats (S7 - S9 Figs); see captions for details of the experimental protocol and how the data were compiled to give relative reelin expression levels for all six animals combined. Assuming a linear relationship between the expression level of reelin and its production rate (τ), and taking advantage of the observation that the relative expression levels of reelin are approximately the same in humans [70], we then used the model to predict the intragroup and intergroup variation in the temporal evolution of [$A\beta_{reelin,L}$].

**Fig 4:**
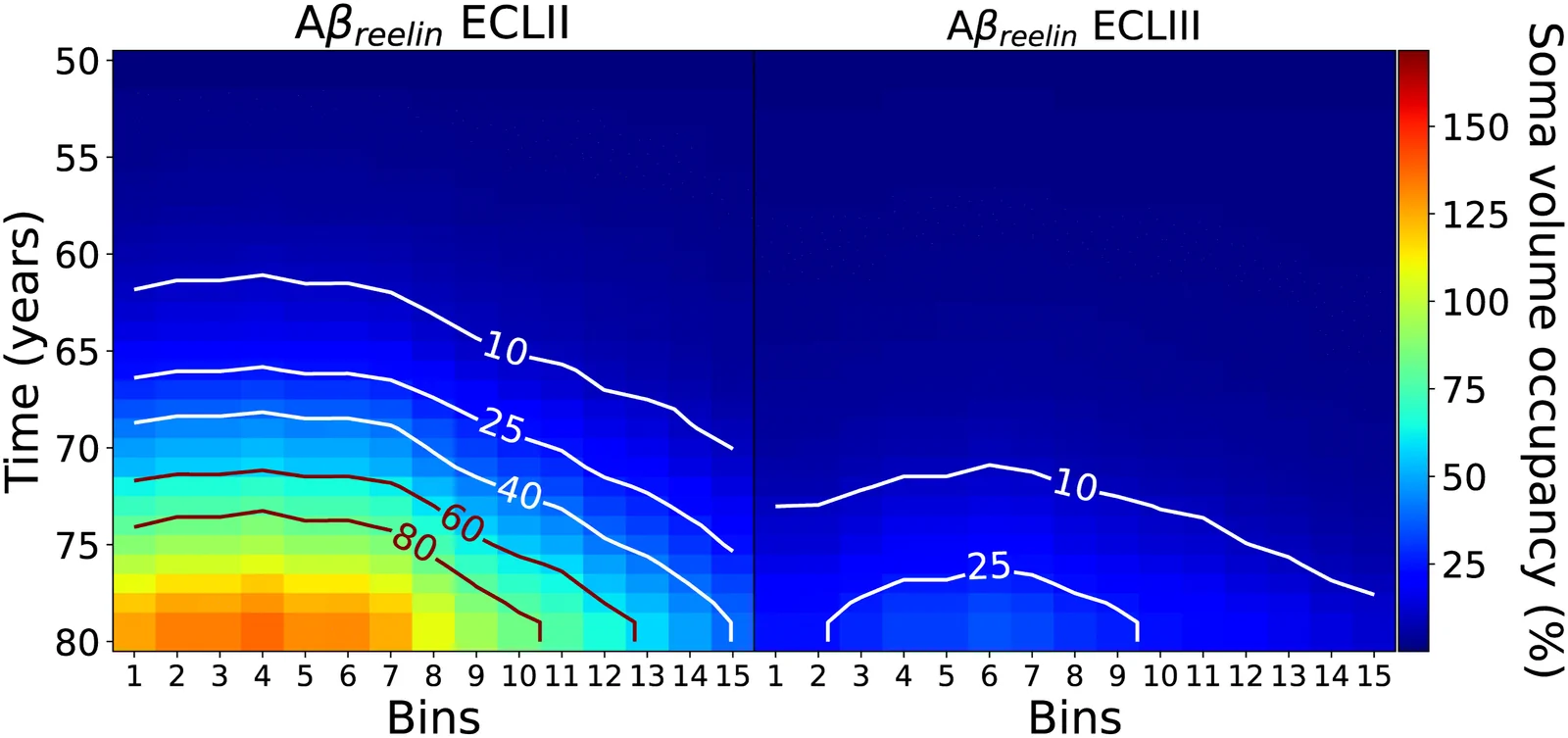
Predicted lateral-to-medial soma volume occupancy of [$A\beta_{reelin,L}$] in ECLII and ECLIII as a function of time. Bin 1 corresponds to the lateral extreme position and increasing numbers refer to increasingly more medial positions. The τ value for Re^+^ neurons used to produce Fig 2 (Table 1) was multiplied with the estimated relative lateral-to-medial reelin expression levels (divided into 15 bins) given in S9 Fig. All parameters were identical to those used to construct Fig 2, except for the variation of τ imposed by the experimental data (S7 - S9 Figs) and letting Ψ = 0.8 at 80 years.

The model predicts patterns of temporal increase in [$A\beta_{reelin,L}$] that are consistent with the empirically observed temporal variation in neuronal cell death in EC [71] (Fig 4). The soma volume occupancy is predicted to reach, for example, 25% in Re^+^ECLII neurons located toward the lateral extreme (i.e., close to the rhinal fissure) several years earlier than those located toward the medial aspect. And the first incidence of 25% soma volume occupancy in Re^+^ECLIII neurons is predicted to occur several years later than in Re^+^ECLII neurons. We acknowledge that this is an idealized calculation that does not account for variation in reelin expression within each of the 15 bins. If we include this variation, we will get a more heterogeneous situation, but the main prediction, concordant with solid empirical data showing that cell death will generally start in ECLII several years before it starts in ECLIII, will not be affected.

### Robustness of the predicted distinct phenotypic difference between Re^+^ECLII and LR neurons

Evidently, the endpoint value of [$A\beta_{reelin,L}$] in Re^+^ECLII neurons depends on the parameters that define the generation of *I*. We quantified this dependency by sampling Ψ, $D_{min}$ and $D_{max}$ from the sets {0.3, 0.4, 0.5, 0.6}, {1, 2, 3, 4} and {5, 10, 15, 20, 25, 30} in a full factorial design and recording the median [$A\beta_{reelin,L}$] value of 101 replicates at ≈72 years. For all Ψ values, there is a nonlinear relationship between the endpoint value of [$A\beta_{reelin,L}$] and $D_{max}$, and $D_{max}$ must be greater than 10–15 days before there is a notable aggregation. A soma volume occupancy of 20% (≈150 μM) at 72 years is obtained when Ψ = 0.5, $D_{max}$ = 25 and $D_{min}$ = 4 (S10 Fig). In LR neurons, the nonlinear relationship is somewhat different, but in this case there is no sign of substantial aggregation for any of the parameter combinations (S11 Fig).

**Fig 5:**
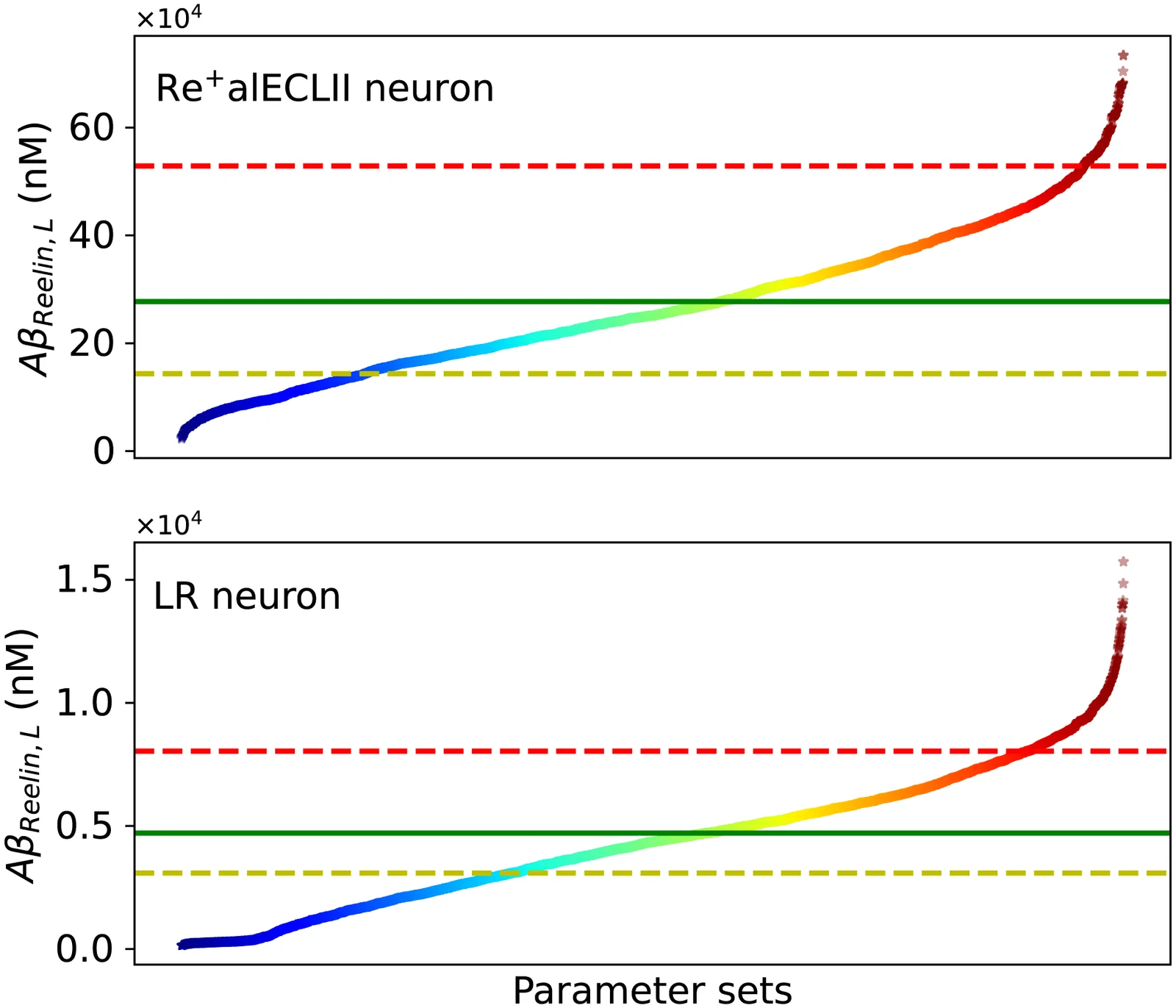
Median endpoint value of [$A\beta_{reelin,L}$] for multiparameter variations centered around the nominal parameter sets for Re^+^ECLII and LR neurons. The parameter sets are sorted in ascending order of their representative endpoint [$A\beta_{reelin,L}$] value. Horizontal lines show the maximum (red dashed lines), median (green solid lines), and minimum endpoint values (yellow dashed lines) of [$A\beta_{reelin,L}$] for the nominal parameter sets. The corresponding soma volume occupancy percentages for the LR and Re^+^ECLII neurons were (0.45, 0.69, 1.2) and (21.1,40.8,77.7), respectively. See main text for further details. We conducted two additional sensitivity analyzes using the open source Python library SALib v 1.47 [72], one estimating how much of the variance of the endpoint value of [$A\beta_{reelin,L}$] could be explained by the variation in each parameter (S12 Fig) and one that assessed the impact of the variation of parameters in Eqs. (5)-(7) on the endpoint [$tau_{p,agg}$] value in Re^+^ECLII neurons (S13 Fig). See captions of S12 and S13 Figs for interpretations of the results.

To study how sensitive the endpoint value of [$A\beta_{reelin,L}$] in the two types of neurons was to the variation of parameters in the dynamic model, we generated 4608 sets of parameters following the Sobol sequence method [73] giving a discrepancy of 0.0007. The parameters $\alpha_{infection}$, β, τ, ρ, δ, $\theta_{1}$, and *T*_1_ varied between ±50% of the nominal values used to generate Figs 1 and 2. We let γ vary between [0.0005−0.05] and *n*_1_ = 3. We then generated 100 different inflammation profiles and Eqs. (1)-(3) were run for each set of parameters and each inflammation profile, resulting in 460,800 independent simulations. For each set of parameters, the endpoint value of [$A\beta_{reelin,L}$] was determined for each inflammation profile. The median value among these 100 values was chosen as the representative [$A\beta_{reelin,L}$] value for that set of parameters. The simulation steps were repeated for the two sets of nominal parameters and the maximum, median and minimum [$A\beta_{reelin,L}$] endpoint values of the 100 simulations were determined. In Re^+^ECLII neurons, the results show that for nearly all parameter sets [$A\beta_{reelin,L}$] reaches very high levels (Fig 5, panel 1). In contrast, in LR neurons (panel 2), almost all parameter sets give rise to a very modest increase in [$A\beta_{reelin,L}$]. This indicates that the predicted marked difference in endpoint [$A\beta_{reelin,L}$] between the two neuron types is quite insensitive to parameter variation.

In the above, because we used fixed parameter values when we run the model, we assessed the robustness of the predicted distinctly different accumulation patterns in Re^+^ECLII and LR neurons by using global sensitivity analysis based on sampling from uniform parameter distributions. An alternative way to make such an assessment is to use specified probability distributions on the parameters instead of fixed parameter values. To test whether such an approach would lead to other conclusions than those reached by the above analysis, we applied a Bayesian Importance Sampling framework adapted from the variance-based global sensitivity analysis detailed by Darges *et al*. [74] aimed at understanding the impact of hyperparameters defining the prior on the posterior statistics of the quantities of interest. This technique relies on an importance sampling distribution to efficiently estimate posterior statistical moments under new target distributions without requiring repeated computationally prohibitive sampling procedures. We exploited this feature by re-weighting our original uniform Sobol sampling space to a posterior likelihood by applying a Gaussian prior centered on the nominal parameter values with a 10% standard deviation. As application of the Gaussian prior centers the probability mass around the baseline by down-weighting the extreme parameter bounds, we expectedly obtained some offset between the probabilistic mean and the deterministic nominal. However, the distinct difference in accumulation patterns between Re^+^ECLII and LR neurons remains (S14 Fig). Thus, at least for this specific case, it seems that the two methods yield results that are entirely consistent with one another.

## Discussion

Although we have succeeded in making quantitative predictions that are clearly in consonance with experimental observations, these predictions depend on several premises, some of which are not yet supported by conclusive experimental data. Of these, the most critical one is evidently the presumption that the A$\beta 42_{reelin}$ complex was the primary component in the soma-filling Aβ42 positive aggregates observed by D’Andrea et al. [7]. However, as already discussed, it is improbable that these lysosomal aggregates were made mainly of Aβ42. Since reelin is the only very large protein known to bind to Aβ42 that has markedly higher expression levels in anterolaterally positioned LII neurons than in most other cortical neurons, it is clearly qualified as a candidate to make sense of the experimental data at hand [7]. Further support comes from the observation that Aβ42 accumulated in an insoluble pool within APP-expressing NT2N neurons differentiated from NT2 cells using retinoic acid (RA), while RA-naïve NT2 cells did not show any accumulation, despite expressing nearly equivalent levels of APP [75]. Since RA-induced differentiation of NT2 cells is accompanied by increased reelin expression [76], this suggests that the observed insoluble pool in NT2N neurons consisted of A$\beta 42_{reelin}$. In other cell culture studies reporting the accumulation of an insoluble Aβ42 pool associated with the EALP [77,78] and where reelin apparently is not expressed at substantial levels, it is likely that the pool consists mainly of Aβ42.

Another critical presumption is the accumulation of aggregation-prone p-tau fragments in Re^+^ECLII neurons (Fig 2), expressed by the function (11) in Eq. (7). Currently, there is no direct experimental evidence for the hypothesized relationship between [$A\beta_{reelin,L}$] and the aggregation rate of tau fragments. The Sobol analysis shows that the endpoint value of [$tau_{p,agg}$] is predominantly sensitive to variation in the threshold value (θ2) (S13 Fig), where [$A\beta_{reelin,L}$] is assumed to begin to severely inhibit the degradation of aggregation-prone truncated forms of p-tau, and to the maximum degradation rate of these forms (ω). Before the appearance of data that could constrain these two parameter values, our quantitative predictions regarding [$tau_{p,agg}$] only show that the hypothesized relationship can in principle significantly contribute to the much earlier appearance of neurofibrillary tangles (NFTs) in Re^+^ECLII neurons than in most other cortical neurons [1,2], since the proaggregation of p-tau fragments will have to precede the appearance of NFTs. Despite the uncertainty attached to these predictions, there is strong experimental support for the notion that the lysosomal surface is an important production site for aggregation-prone p-tau fragments [61]. Thus, there is ample reason to suggest that enhanced production may arise from an increase in residence time of these fragments on the lysosomal surface and that this increase can be directly related to an extreme supraphysiological increase in [$A\beta_{reelin,L}$].

Due to the paucity of data, we used a 1:1 stoichiometry for A$\beta 42_{reelin}$. It can very well be that several Aβ42 molecules are bound to each reelin molecule, which would affect the dynamics. We therefore tested the impact a 3:1 stoichiometry would have on the main predictions by revising Eqs. (1)-(3) accordingly (see S15 Fig caption for details). Since it is reasonable to demand that the immune response times should be similar in the two cases, we imposed the restriction that the time it takes for [$GSK3\beta_{p}$] to reach the 10% level after a sudden rise in Aβ42 production should be approximately the same (≤2%) as for the case of 1:1 stoichiometry and that the immune response behavior should otherwise be qualitatively similar to the behavior of the original model (S5 and S6 Figs). The 10% level constraint and the behavioral constraint were met by tripling the value of $\alpha_{infection}$ in both LR and Re^+^ ECLII neurons and letting $5\cdot 10^{-7}\leq \gamma \leq 5\cdot 10^{-12}$. With this parameterization, the long-term predictions of the revised model were similar to those of the original model except that [$A\beta_{L}$] shows periods of temporal increase and that levels are higher in both neuron types (S15 and S16 Figs). Since the pronounced end-point phenotypic difference between LR and Re^+^ ECLII neurons is maintained, the use of an experimentally unjustified 1:1 stoichiometry does not appear to pose a major concern regarding the main conclusions of this study. New experimental data are needed before this issue can be further considered.

In addition to the above three potential issues, several of the parameter values are ballpark estimates because of the paucity of experimental data. However, it should be noted that only Eqs. (1)-(3) are needed to describe the evolution of [$A\beta_{reelin,L}$], and all their parameter values, except those in functions (8) and (10), were used to describe the dynamics of Aβ42, reelin and A$\beta 42_{reelin}$ in native physiology [13]. Thus, they were deliberately not tuned to accommodate the quantitative correspondence between the predicted soma volume occupancy of [$A\beta_{reelin,L}$] and the experimental data (Fig 2).

We deliberately addressed only the dynamics associated with Aβ42. This is of course a crude simplification as other Aβ variants are likely to be part of [$A\beta_{reelin,L}$] and [$A\beta_{L}$]. However, given the particular importance of Aβ42 in the development of AD, we consider this to be justifiable, especially as long as data that would allow increasing the resolution of the model are lacking.

D’Andrea et al. [7] provided ample evidence for neuronal lysis as a source of plaques in EC. More recent findings in mice [10] indicating that cellular lysis can be a generic mechanism of plaque formation support this, although the matter is still debated. Albeit we do not know what the main triggers for neuronal lysis are, there is a well-documented strong link between the presence of p-tau oligomers and mitochondrial dysfunction [79]. And Re^+^ECLII neurons are clearly more prone to experience mitochondrial impairment very early in disease development than LR neurons [80,81]. One possible explanation for this is that since Re^+^ECLII neurons appear to have a very high energy demand [82], they could be particularly vulnerable to a drop in energy supply. Our model predictions are clearly consistent with this scenario and the observation that NFTs typically first appear in Re^+^ECLII neurons. However, our results are also consistent with the notion that a very high occupancy of the soma volume by EALP cargo can by itself trigger a cascade of pathophysiological processes, including mitochondrial dysfunction and formation of p-tau oligomers, which eventually lead to cell death.

If we assume that neuronal lysis is a factor behind plaque generation in EC, we are confronted with the puzzle that although Re^+^ECLII neurons accumulate a conspicuously high amount of intraneuronal Aβ42, ECLII forms far fewer plaques compared to the deeper layers of EC as AD progresses. Considering that Re^+^ECLII neurons may start to disappear prior to the onset of mild cognitive impairment, a possible explanation is that dead cells, including those that have been lysed and may have formed transient plaques, are removed relatively quickly in the very early phase of AD development. This conception is supported by the observation that dead neurons are removed quite quickly by concerted operation of astrocytes and microglia [83], and that microglia are capable of completely removing amyloid aggregates over a few weeks by digestive exophagy [84]. Since our results indicate that [$A\beta_{reelin,L}$] reaches critical levels in Re^+^ECLII neurons several years before Re^+^ECLIII neurons (Fig 4), this suggests that the fully operational astrocyte/microglia system that is likely to exist in the very early phase of disease development has the ability to remove cells that have been lysed without leaving behind a large number of plaques. However, as the disease progresses, this system will probably be prone to saturation effects and other types of dysfunctionalization.

## Conclusion

Based on the premise that reelin functions as a sink for Aβ42, we have shown that it is possible to sensibly project the decades-long effects of recurrent short-term senescence-related inflammation on the development of a major neurological disorder at the molecular level that are consistent with key experimental data and provide multiple experimentally testable predictions. Although this is an important conceptual advance, our model should be looked upon as a first step towards a genuine representation of the dynamics causing the EC to be a cradle for AD. However, its capacity to make sound quantitative predictions indicates that such a representation is within reach if its construction is given sufficient theoretical-experimental attention.

## Supporting information

S1 FigDistribution of inflammation events over the period [$\Omega_{1},\Omega_{2}$] years.The distribution was generated by dividing the period into the corresponding number of months ($N_{m}$). We then defined an inflammation propensity function $P(t),t\in \{1,2,...,N_{m}\}$. For simplicity, we let *P*(*t*) increase linearly, with *P*(1)=0 and $P(N_{m})=\Psi$. We then sampled, for each month, a random number from X~U(0,1). If the number was ≤P(t), then the neuron experienced an inflammation event starting on the first day of the month, otherwise not. The duration of an inflammation period was allowed to be $X\sim U(D_{min},D_{max})$, where $D_{min}$ and $D_{max}$ denote the minimum and maximum number of days that the inflammation event could last. The above procedure generated a vector (*I*) consisting of time-labeled elements describing when and for how long a neuron was exposed to an inflammation event during the period [$\Omega_{1},\Omega_{2}$]. The parameter values used to generate the inflammation event vector *I* were: $\Omega_{1}$ = 50 years, $\Omega_{2}$ = 72 years, Ψ = 0.6, $D_{min}$ = 5 days, $D_{max}$ = 29 days. This particular realization of *I* had 74 inflammation events with a mean duration of 16.8 days.(TIF)

S2 FigExamples of function (8) ($H_{1}([A\beta ],\theta_{1},n_{1})$) that is part of Eqs. (3), (4) and (7).The blue line refers to a first order process, i.e., the degradation rate is given by the specific decay constant times [Aβ]. Since we assume the existence of a moderately sigmoidal dose-response relationship, we chose to use the green inverse Hill function graph with *n*_1_ = 3. $\theta_{1}$ = 2.36x10^5^ molecules, i.e., 80% of the maximal copy number of free Aβ42 during an inflammation event when *H*_1_(.) is set to zero.(TIF)

S3 FigSmooth version of the threshold function (10) ($S_{0}^{T_{1}}([A\beta_{reelin,L}]$) used in Eq. (3).The function (red graph) expresses that as long as [$A\beta_{reelin,L}$] is below *T*_1_, the degradation rate is according to a first order process (δ [$A\beta_{reelin,L}$]). When it is above, the degradation kinetics becomes zero order due to saturation of the degradation machinery. Since this function is not smooth as required by the numerical solver, in the simulations we instead used the smooth version: $S_{0}^{T_{1}}([A\beta_{reelin,L}],\epsilon )=\frac{1}{2}\left\{T_{1}+([A\beta_{reelin,L}{]}^{2}+\epsilon^{2}{)}^{\frac{1}{2}}-{\left({\left([A\beta_{reelin,L}]-T_{1}\right)}^{2}+\epsilon^{2}\right)}^{\frac{1}{2}}\right\}$. The blue graph depicts $\delta S_{0}^{T_{1}}([A\beta_{reelin,L}],\epsilon )$ with ϵ = 1*x*10^5^ to make the graphs easily distinguishable. In the simulations, we let ϵ = 1, making the graphs indistinguishable. The values of δ and *T*_1_ are given in Table 1 in the main text. The function (11) ($S_{0}^{T_{2}}([A\beta_{L}]$) was smoothed in the same way.(TIF)

S4 FigExamples of function (9) ($H_{2}([A\beta_{reelin,L}],\theta_{2},n_{2})$) that is part of Eq. (7).The blue line refers to a first order process, i.e., the degradation rate is given by ω[$A\beta_{reelin,L}$]. $\theta_{2}$ = 1x10^8^. Since we assumed the existence of a moderate sigmoidal dose-response relationship, we chose to use the green inverse Hill function graph with *n*_2_ = 3.(TIF)

S5 Fig Short-term dynamic response to a temporary inflammation event in an LR neuron. The parameter values are given in Table 1 in the main text.
(TIF)

S6 Fig Short-term dynamic response to a temporary inflammation event in a Re^+^ECLII neuron. Parameter values are given in Table 1 in the main text.
(TIF)

S7 Fig Reelin expression gradient in ECLII reelin expressing neurons in wild type Wistar rats. ECLII reelin signal intensity was measured from the lateral extreme (bordering the rhinal fissure) and successively more medial positions, up to and including the point at which the signal has the lowest intensity, which corresponds approximately to the border that separates the cytoarchitectonically defined lateral entorhinal cortex from the medial entorhinal cortex. The measurements were segregated into 15 equally sized bins. Methods in brief: Transcardial perfusions and tissue processing were done as described [20]. Immunolabeling (40 um sections) included anti-reelin (1:1000; G10 clone; Merk, Cat# MAB5364; RRID: AB_2179313) with secondary antibody Alexa 635 Goat anti-mouse (1:500; ThermoFisher, Cat# A-31574). Densitometric measurements of reelin signal were done in QuPath (version 0.5.0), where we delineated ECLII and calibrated the ‘cell detection’ feature to detect reelin expressing neuronal somata. We used six animals (three 3-month-olds (IDs: 16235, 16491, 17709) and three 12-month-olds (IDs: 17710, 26347, 26760)). From each animal, to get optimal coverage of the lateral-to-medial extent, we used seven roughly equally spaced sections contained within Bregma -6.12 mm to -8.16 mm.
(TIF)

S8 Fig Lateral-to-medial reelin signal intensity in ECLIII of 3 (IDs: 16235, 16491, 17709) or 12 (IDs: 17710, 26347, 26760) month old wild type rats. Each data point is the mean value of 7 tissue slices, normalized by the maximum signal value across the 15 bins. Experimental protocol identical with that of ECLII (see caption to S7 Fig).
(TIF)

S9 Fig Lateral-to-medial reelin signal intensity in ECLII and ECLIII. Panels 1 and 2: Normalized lateral-to-medial reelin signal intensity across the 15 bins in ECLII and ECLIII averaged over all seven tissue sections and all six animals. Panel 3: The lateral-to-medial reelin signal intensity ratio of ECLII and ECLIII averaged over all seven tissue sections and all six animals.
(TIF)

S10 FigThe impact of varying the parameters Ψ, $D_{min}$ (see legend panel 1 (top left) and $D_{max}$ (see x-axis tick labels) underlying the inflammation event vector *I* on the endpoint concentration of [$A\beta_{reelin,L}$] in Re^+^ECLII neurons.(TIF)

S11 FigThe impact of varying the parameters Ψ, $D_{min}$ (see legend panel 1 (top left) and $D_{max}$ (see x-axis tick labels) underlying the inflammation event vector *I* on the maximum value of [$A\beta_{reelin,L}$] in LR neurons.(TIF)

S12 FigSobol indices associated with [$A\beta_{reelin,L}$].Sobol first order (S1) and total order (ST) indices with 95% confidence intervals for [$A\beta_{reelin,L}$] endpoint value-related parameters of LR and Re^+^ECLII neurons [73], using the open source Python library SALib v 1.47 [72]. The S1 index describes the influence of a specific parameter on the endpoint value of [$A\beta_{reelin,L}$] when all other parameter values remain at their nominal values. The ST index describes the influence of a given parameter and its interactions with other parameters. The analysis suggests that in LR neurons, the variation in $\alpha_{infection}$ has a much larger impact than τ and the degradation parameters β, δ, $\theta_{1}$ and *T*_1_ (S7 Fig, left panel column), while in Re^+^ECLII neurons, the endpoint value of [$A\beta_{reelin,L}$] is more sensitive to the variation in τ, δ, and *T*_1_ (S9 Fig, right panel column). This implies that the predicted increase in [$A\beta_{reelin,L}$] can be directly related to the observation that the reelin production rate is much higher in Re^+^ECLII neurons than in LR neurons [13], and that it depends greatly on the degradation dynamics of [$A\beta_{reelin,L}$]. That is, either the degradation rate must be very low in general (δ) or the degradation machinery becomes saturated above a given concentration (*T*_1_).(TIF)

S13 FigSobol indices associated with [$tau_{p,agg}$].Sobol first order (S1) and total order (ST) indices with 95% confidence intervals for [$tau_{p,agg}$] endpoint concentration-related parameters of LR and Re^+^ECLII neurons. The parameters of Eqs. (1)-(3) were fixed at their nominal values, Eq. (4) was ignored, and the parameter ϕ in Eq. [7] was fixed to 1.0. *n*_2_ was fixed to 3, while the remaining seven parameters in Eqs. (5)-(7) were allowed to vary between ± 50% of their nominal values. The number of generated parameter sets was 4608, with a discrepancy of 0.0007. The analysis followed the same procedure as described for S12 Fig. The Sobol analysis shows that the [$tau_{p,agg}$] endpoint value is predominantly sensitive to variation in the threshold value (θ2) where accumulated $A\beta 42_{reelin}$ is assumed to begin to severely inhibit the degradation of aggregation-prone truncated forms of p-tau.(TIF)

S14 FigAssessment of the impact of using Gaussian-distributed parameter values.To assess the effects on the robustness of our results using normal-distributed parameter values instead of fixed values on predicted end-point values [$A\beta_{reelin,L}$] and [$tau_{p,agg}$] in Re^+^ ECLII and LR neurons, we applied a Bayesian Importance Sampling framework adapted from the variance-based global sensitivity analysis detailed by Darges et al. [74]. This technique relies on an importance sampling distribution to efficiently estimate posterior statistical moments under new target distributions without requiring repeated, computationally prohibitive sampling procedures. Here, the original uniform Sobol sampling space (grey dashed lines) was re-weighted to a posterior likelihood (solid line) by applying a Gaussian prior centered on the nominal parameter values with a 10% standard deviation. **a**, **c**, Probability densities of maximum [$A\beta_{reelin,L}$] in Re^+^ ECLII and LR neurons and comparison of the two predicted mean values. **b**, **d**, Probability densities of maximum [$tau_{p,agg}$] in Re^+^ ECLII (b) and LR (d) neurons and comparison of the two predicted mean values.(TIF)

S15 FigPredicted time evolution of four of the seven state variables in an LR neuron over 22 years when using a 3:1 $A\beta_{reelin}$ stoichiometry.The first three equations of the original model were revised to accommodate for a 3:1 stoichiometry, following standard rules for mass action kinetics: $\frac{d[A\beta ]}{dt}=\alpha (t)-\beta [A\beta ]-3\cdot \gamma [A\beta {]}^{3}[Reelin],$$\frac{d[Reelin]}{dt}=\tau -\rho [Reelin]-\gamma [A\beta {]}^{3}[Reelin],$$\begin{array}{c} \frac{d[A\beta_{reelin,L}]}{dt}=\gamma [A\beta {]}^{3}[Reelin]-\delta H_{1}([A\beta ],\theta_{1},n_{1})S_{0}^{T_{1}}([A\beta_{reelin,L}]), \end{array}$ See Fig 1 caption and text in Discussion for further explanation.(TIF)

S16 FigPredicted time evolution of four of the seven state variables in a Re^+^ECLII neuron over 22 years when using a 3:1 $A\beta_{reelin}$ stoichiometry.See the captions of Figs 2 and S15 and the text in the Discussion for further explanation.(TIF)

S1 Appendix Appendix. (PDF)
