# Supplementary material for: High reelin expression may explain why a subgroup of entorhinal cortex neurons functions as an initial nucleation site of Alzheimer’s disease
Source: PLoS Comput Biol. 2026 Aug 25;22(8):e1014532. doi: 10.1371/journal.pcbi.1014532 (PMC13506104; doi:10.1371/journal.pcbi.1014532)
Supplement: Supplementary file 1 [file pcbi.1014532.s017.pdf]

S1 Appendix

High reelin expression may explain why a subgroup of entorhinal cortex neurons functions as an initial nucleation site of Alzheimer's disease

Asgeir Kobro-Flatmoen[1,2,*], Jagir R. Hussan[3], Peter J. Hunter[3], Stig W. Omholt[1,2]

1 Kavli Institute for Systems Neuroscience, Norwegian University of Science and Technology (NTNU), Trondheim, Norway
2 Norwegian Health Association Centre for Dementia Research, Norwegian University of Science and Technology (NTNU), Trondheim, Norway
3 Auckland Bioengineering Institute, The University of Auckland, Auckland, New Zealand

* asgeir.kobro-flatmoen@ntnu.no

Data motivating the parameterization of the inflammation vector *I(t)*

It is well known that several common infections and certain invasive medical procedures that trigger systemic inflammation also cause secondary neuroinflammation, and that bouts of neuroinflammation tend to become more detrimental to brain health as we age [1]. To arrive at a reasonable and conservative estimate of the number of times a person between the ages of 50-72 years experiences a neuroinflammatory episode as a result of systemic inflammation, we used statistics from the US Centers for Disease Control and Prevention (CDC) along with data from several published papers. In the following, we provide estimates for the incidence rates of 12 of the most common causes of systemic inflammation in the relevant age range of 22 years. Causes are listed in order of estimated prevalence. We recognize that several of the causes of systemic inflammation listed below will influence each other and that this likely gives rise to complex interactions that cannot be captured by a single number. However, for the purpose of reaching a conservative estimate, we think that our approach is justifiable.

Exposure to pollution and toxins

Environmental pollution is a major contributor to neuroinflammation, and is likely to disproportionately affect elderly people with a compromised blood-brain barrier [2, 3]. People in urban areas will experience several bouts of systemic inflammation per year, mainly due to air pollution [4, 5]. Given that 80% of all people in the western world live in cities (UN_urban), we can safely assume that people will, on average, experience more than one bout of systemic inflammation due to exposure to pollution and toxins each year. We therefore, conservatively, set this number at 1.1 bouts / year, giving an estimate of 24 bouts of systemic inflammation due to exposure to pollution and toxins within our age range.

Sleep deprivation

There are convincing data to show that sleep deprivation induces neuroinflammation [6]. More than a third of people aged 50 to 72 years are sleep deprived according to the

CDC (that is, less than 7 hours of sleep per day) (CDC_sleep). Although person-to-person variation is likely to be high, we believe it is safe to assume that a person between the ages of 50-72 experiences on average at least one bout of substantial sleep deprivation per year. Thus, we reach 22 neuroinflammatory bouts caused by sleep deprivation in this age range.

Vaccines

Although vaccines rarely cause serious complications and have saved ≈154 million lives in the last 50 years alone [7], they cause an inflammatory response that is likely to include a neuroinflammatory component [8]. The recommended schedule for vaccination for people over 50 years old in the US, which is similar to that in most other countries in the western world, includes an annual influenza vaccine, at least two COVID-19 vaccines, plus other vaccinations against common pathogens such as, for example, diphtheria, tetanus, and whooping cough (CDC_vaccines). However, since not all people follow the recommended vaccination schedule, we conservatively estimate the number of neuroinflammation bouts due to vaccinations received between the ages of 50-72 to be 10.

Allergic rhinitis (a.k.a. hay fever)

Allergic rhinitis involves a systemic inflammatory reaction and, although many details must be worked out, such reactions also include a substantial neuroinflammatory component [9]. Data from the US show that about 7.8% of people have allergic rhinitis and that these people will experience between 3-10 inflammatory bouts each year; taking a conservative approach, we assumed that these people will experience 4 bouts. Thus, taking the number of people in the U.S. around time these statistics were collected (330 million) we may calculate that, on average, a person will experience 6.86 bouts of neuroinflammation due to allergic rhinitis over a 22-year period. Several other less common allergic reactions are also likely to result in a systemic (and hence neuro) inflammatory reaction. However, since we do not have data on their combined prevalence, and in the vein of aiming for a conservative estimate, we have not included these here.

Major surgery

Major surgery induces systemic inflammation that is generally proportional to the invasiveness of the procedure [10–13]. Data from CDC, available for the year 2010 (CDC_surgery; see also CDC_procedures), show that the number of major surgeries in the U.S. population older than 45 years was 18.67 million. Since the number of people over 45 years of age in the US is 139 million, the average person is estimated to experience 2.86 bouts of neuroinflammation due to surgery over our target 22-year period.

Influenza

The CDC estimates that, in the US, the number of influenza cases per year ranges from 9.3 to 41 million (CDC_fluburden). Using the mean (25 million), and with a US population of about 330 million at the time when these statistics were collected, the average person is estimated to experience 1.67 bouts of neuroinflammation due to influenza over a 22-year period.

Norovirus and other gastrointestinal diseases

Based on CDC data (CDC_norovirus), about 20 million US citizens get infected by norovirus per year, while about 4.4 million citizens will suffer at least one bout of some other gastrointestinal illnesses per year. With a population of about 330 million at the time when these statistics were collected, the average person may be expected to experience 1.6 bouts of neuroinflammation due to such illnesses over a 22-year period.

Respiratory Syncytial Virus (RSV)

RSV tends to cause serious inflammation in small children and older adults, and has an annual incidence rate for the elderly of about 7% [14]. Between ages 50-72 we can thus calculate that, on average, an individual will experience (22 x 0.07) 1,54 bouts of RSV.

Parainfluenza Virus

Parainfluenza virus is known, among other conditions, to cause bronchitis and pneumonia in older adults [15]. CDC data show that the annual U.S. incidence rate is $\approx$5% (CDC_incidence), which for a population of 330 million gives an estimate of 1.1 neuroinflammatory bouts over the 22-year period.

Urinary tract infection

Excluding the childhood period, biomarker data from the US and Canada indicate that women will experience at least 1-2 bouts of urinary tract infections over a lifetime, while for men the number is less than 1. However, the number of self-reported incidents is much higher [16]. Based on these data, we assumed one bout of neuroinflammation due to urinary tract infection during the 22-year period.

Human Metapneumovirus (hMPV)

Like parainfluenza virus, hMPV is a common virus that tends to cause serious inflammation in small children and older adults [17]. CDC data show that the annual incidence rate is $\approx$3.8% (CDC_hMPV). This gives an estimate of 0.84 neuroinflammatory bouts over the 22-year period. Other common upper respiratory tract infections involve, for example, adenovirus (annual incidence rate of about 3%) and rhinovirus (annual incidence rate of $\approx$20%) (CDC_rhino). Since these are believed to only rarely cause a substantial inflammatory response, we have chosen to exclude them from the estimate.

Inflammatory Bowel Disease (IBD)

The US incidence rate of IBD in subjects older than 50 years is $\approx$1% [18], giving an average estimate of 0.2 bouts of neuroinflammation due to IBD over the 22-year period.

Conclusion

Our estimates apply to people in the Western world, although they are likely to be also relevant to people in many nonwestern countries. Based on data on the 12 sources of systemic inflammation listed above, we find that, conservatively estimated, a person between ages 50-72 will experience on average more than 70 bouts of systemic inflammation that is likely to lead to secondary neuroinflammation in individuals in the given age range. Due to the lack of quantitative data, we have not included neuroinflammation events caused by poor oral health and changes in the gut

microbiome [1]. Furthermore, in senescent physiology, recurrent inflammation events of peripheral and central nervous system origin and of variable duration may be caused by several other processes in addition to those listed here [19–23]. In line with the above considerations, the inflammation vector *I(t)* depicted in **S1 Fig** contains 74 inflammation events of varying duration. Due to stochasticity, this number will vary somewhat for each realization of *I(t)*, but will overall be in the range of 65-80 events. Unfortunately, we have not found any data that can be used to constrain the parameter values that define the mean length and the variability of the duration of an inflammation event. In **S1 Fig**, the mean duration is 16.8 days. This may be an overestimate, but considering that the total number of inflammation events may very well be higher than what we have stipulated, allowing the mean duration to be reduced, we think that the parameterization of *I(t)* used generates a family of inflammation profiles that are physiologically sound.

References

1. Heneka MT, van der Flier WM, Jessen F, Hoozemanns J, Thal DR, Boche D, et al. Neuroinflammation in Alzheimer Disease. Nat Rev Immunol. 2025 May;25(5):321-52. doi:10.1038/s41577-024-01104-7.

2. Block ML, Calderón-Garcidueñas L. Air Pollution: Mechanisms of Neuroinflammation and CNS Disease. Trends Neurosci. 2009 Sep;32(9):506-16. doi:10.1016/j.tins.2009.05.009.

3. Costa LG, Cole TB, Dao K, Chang YC, Coburn J, Garrick J. Chapter Seven - Neurotoxicity of Air Pollution: Role of Neuroinflammation. In: Aschner M, Costa LG, editors. Advances in Neurotoxicology. vol. 3 of Role of Inflammation in Environmental Neurotoxicity. Academic Press; 2019. p. 195-221. doi:10.1016/bs.ant.2018.10.007.

4. Meng X, Liu C, Chen R, Sera F, Vicedo-Cabrera AM, Milojevic A, et al. Short Term Associations of Ambient Nitrogen Dioxide with Daily Total, Cardiovascular, and Respiratory Mortality: Multilocation Analysis in 398 Cities. BMJ. 2021 Mar:n534. doi:10.1136/bmj.n534.

5. Thompson AMS, Zanobetti A, Silverman F, Schwartz J, Coull B, Urch B, et al. Baseline Repeated Measures from Controlled Human Exposure Studies: Associations between Ambient Air Pollution Exposure and the Systemic Inflammatory Biomarkers IL-6 and Fibrinogen. Environ Health Perspect. 2010 Jan;118(1):120-4. doi:10.1289/ehp.0900550.

6. Cao D, Zhao Y, Wang Y, Wei D, Yan M, Su S, et al. Effects of Sleep Deprivation on Anxiety-Depressive-like Behavior and Neuroinflammation. Brain Res. 2024 Aug;1836:148916. doi:10.1016/j.brainres.2024.148916.

7. Shattock AJ, Johnson HC, Sim SY, Carter A, Lambach P, Hutubessy RCW, et al. Contribution of Vaccination to Improved Survival and Health: Modelling 50 Years of the Expanded Programme on Immunization. The Lancet. 2024 May;403(10441):2307-16. doi:10.1016/S0140-6736(24)00850-X.

8. Nath A. Neurologic Complications With Vaccines. Neurology. 2023 Oct;101(14):621-6. doi:10.1212/WNL.0000000000207337.

9. Konstantinou GN, K Gerasimos N, K Christopher, P Konstantinos, and Makris M. Further Understanding of Neuro-Immune Interactions in Allergy:

Implications in Pathophysiology and Role in Disease Progression. J Asthma Allergy. 2022 Jan;15:1273-91. doi:10.2147/JAA.S282039.

10. Watt DG, Horgan PG, McMillan DC. Routine Clinical Markers of the Magnitude of the Systemic Inflammatory Response after Elective Operation: A Systematic Review. Surgery. 2015 Feb;157(2):362-80. doi:10.1016/j.surg.2014.09.009.

11. Lindholm EE, Aune E, Seljeflot I, Otterstad JE, Kirkebøen KA. Biomarkers of Inflammation in Major Vascular Surgery: A Prospective Randomised Trial. Acta Anaesthesiol Scand. 2015 Jul;59(6):773-87. doi:10.1111/aas.12466.

12. Bain CR, Myles PS, Martin C, Wallace S, Shulman MA, Corcoran T, et al. Postoperative Systemic Inflammation after Major Abdominal Surgery: Patient-Centred Outcomes. Anaesthesia. 2023;78(11):1365-75. doi:10.1111/anae.16104.

13. Lassig AAD, Lindgren BR, Itabiyi R, Joseph AM, Gupta K. Excessive Inflammation Portends Complications: Wound Cytokines and Head and Neck Surgery Outcomes. The Laryngoscope. 2019;129(7):E238-46. doi:10.1002/lary.27796.

14. Kim T, Choi SH. Epidemiology and Disease Burden of Respiratory Syncytial Virus Infection in Adults. Infect Chemother. 2024 Mar;56(1):1-12. doi:10.3947/ic.2024.0011.

15. Pawełczyk M, Kowalski ML. The Role of Human Parainfluenza Virus Infections in the Immunopathology of the Respiratory Tract. Curr Allergy Asthma Rep. 2017 Mar;17(3):16. doi:10.1007/s11882-017-0685-2.

16. Foxman B. The Epidemiology of Urinary Tract Infection. Nat Rev Urol. 2010 Dec;7(12):653-60. doi:10.1038/nrurol.2010.190.

17. Shafagati N, Williams J. Human Metapneumovirus - What We Know Now. F1000Res. 2018 Feb;7:135. doi:10.12688/f1000research.12625.1.

18. Lewis JD, Parlett LE, Jonsson Funk ML, Brensinger C, Pate V, Wu Q, et al. Incidence, Prevalence, and Racial and Ethnic Distribution of Inflammatory Bowel Disease in the United States. Gastroenterology. 2023 Nov;165(5):1197-205.e2. doi:10.1053/j.gastro.2023.07.003.

19. Franceschi C, Garagnani P, Parini P, Giuliani C, Santoro A. Inflammaging: A New Immune–Metabolic Viewpoint for Age-Related Diseases. Nat Rev Endocrinol. 2018 Oct;14:576-90. doi:10.1038/s41574-018-0059-4.

20. Furman D, Campisi J, Verdin E, Carrera-Bastos P, Targ S, Franceschi C, et al. Chronic Inflammation in the Etiology of Disease across the Life Span. Nat Med. 2019;25:11. doi:10.1038/s41591-019-0675-0.

21. Walker KA, Ficek BN, Westbrook R. Understanding the Role of Systemic Inflammation in Alzheimer's Disease. ACS Chem Neurosci. 2019 Aug;10:3340-2. doi:10.1021/acschemneuro.9b00333.

22. Zhang W, Xiao D, Mao Q, Xia H. Role of Neuroinflammation in Neurodegeneration Development. Sig Transduct Target Ther. 2023 Jul;8(1):1-32. doi:10.1038/s41392-023-01486-5.

23. Mekhora C, Lamport DJ, Spencer JPE. An Overview of the Relationship between Inflammation and Cognitive Function in Humans, Molecular Pathways and the Impact of Nutraceuticals. Neurochem Int. 2024 Dec;181:105900. doi:10.1016/j.neuint.2024.105900.
