# Supplementary material for: High reelin expression may explain why a subgroup of entorhinal cortex neurons functions as an initial nucleation site of Alzheimer’s disease
Source: PLoS Comput Biol. 2026 Aug 25;22(8):e1014532. doi: 10.1371/journal.pcbi.1014532 (PMC13506104; doi:10.1371/journal.pcbi.1014532)
Supplement: Supplementary file 2 [file pcbi.1014532.s019.pdf]

Response to Reviewers

Reviewer #1

1. *The authors develop a computational model that uses high levels of reelin in ECLII neurons to explain high levels of AB42 in the same cells that would be otherwise difficult to explain. The importance is that ECLII has been implicated as a birthplace of Alzheimer's disease (AD) progression, so the mechanistic model advanced potentially captures/explains the earliest of AD cascading dynamics. The model is an ODE model that captures the effect of intermittent, short-term increased production of AB42 and is driven by a random vector of inflammation events increasing production of AB42. The paper is well written and the modeling decisions carefully follow experimental data and previous studies in the AD literature.*

Response: We would like to thank the reviewer for his positive evaluation of the paper and for pressing us on why we did not pursue a more Bayesian-inspired modeling approach.

2. *On the other hand, the model builds on work recently published in the journal [14]. The specific modeling contribution seems to be the careful extension of a previous model—implicating reelin levels in AB42 growth over a time frame of hours for healthy(?) neurons—to a time frame of 22 years for senescent neurons. It would be helpful if the authors could more clearly delineate the work's contribution with respect to [14].*

Response: We have revised the first paragraph in the subsection "The dynamic model" to better delineate the two models.

3. *While the model parameter specifications are well-articulated, I question the use of fixed parameter values. It would make more sense to specify probability distributions on parameter values. This would provide a better idea of the range of possible outcomes and robustness of the model. I would hope to see this point seriously attended to in a revision.*

Response: With regard to range of possible outcomes and robustness of the model, we are not sure we understand why it would make more sense to specify probability distributions on parameter values instead of using fixed values accompanied by a thorough global sensitivity analysis. Some of our parameters can be reasonably well constrained, others not (due to paucity of guiding data). And in none of the cases do we have data supporting a particular choice of distribution. Even if we introduced hypothetical distributions, the prior mean values would still be the values we have used in the study. The main purpose of the global sensitivity analysis was to get a measure on the range of possible outcomes and thus a measure of how robustly our model predicts distinctly different phenotypes in high and low reelin neurons after 22 years of exposure to recurrent inflammation bouts. By sampling parameter values from uniform distributions with quite wide margins, we confirm that our main predictions are pretty robust to parameter variation.

However, we agree that we should have addressed the alternative approach suggested by the reviewer. To this end we have, at the end of the subsection "Robustness of the predicted distinct phenotypic difference between Re^{+}ECLII and LR neurons" included a new paragraph, and we have included a new Supplementary Figure 14 showing a quantiative comparison of the two approaches.

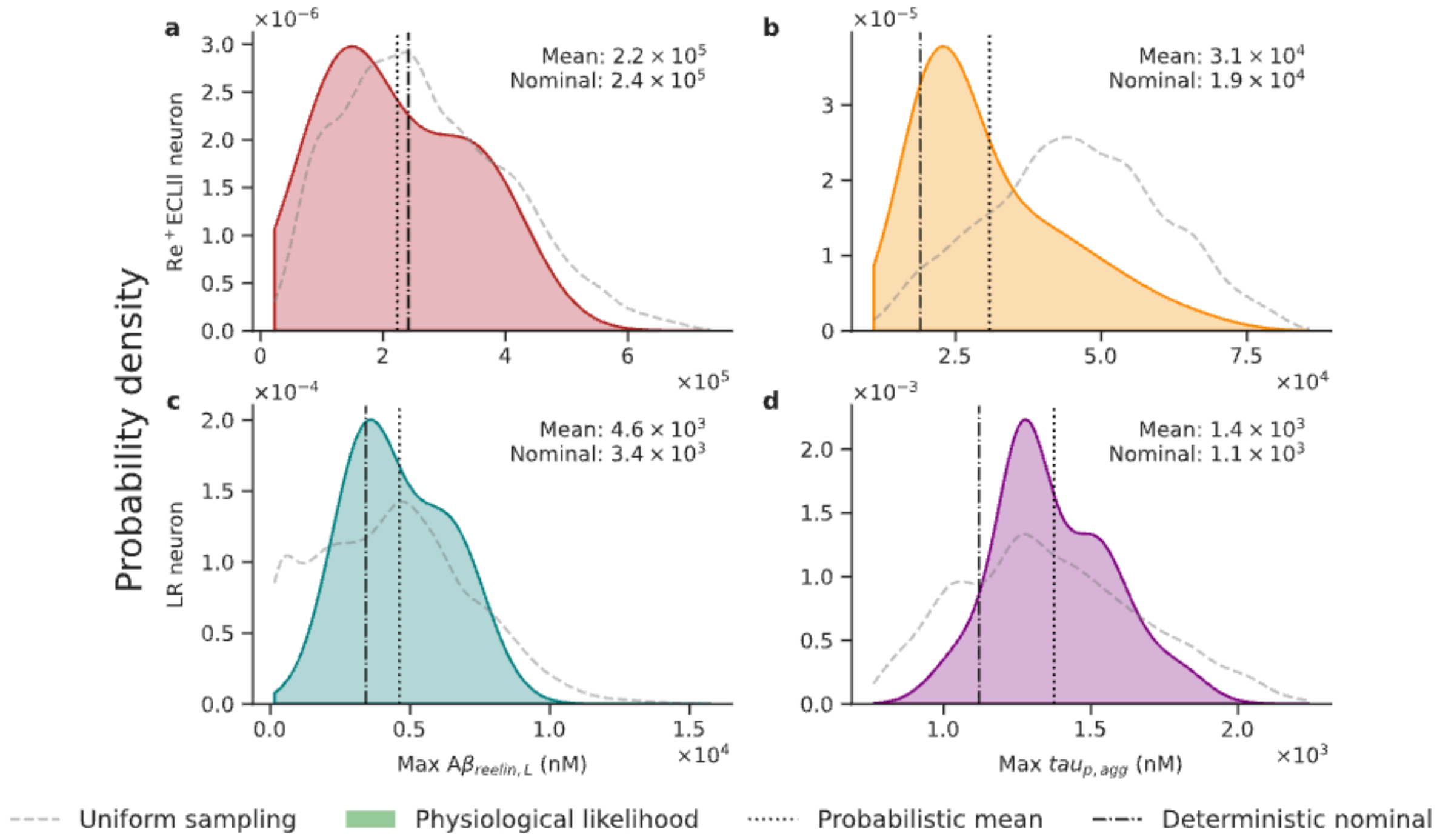

Fig. S14 **Assessment of the impact of using Gaussian-distributed parameter values**. To assess the effects on the robustness of our results using normal-distributed parameter values instead of fixed values on predicted end-point values $A\beta_{reelin,L}$ and $tau_{p,agg}$ in Re+ ECLII and LR neurons, we applied a Bayesian Importance Sampling framework adapted from the variance-based global sensitivity analysis detailed by Darges et al.[74]. This technique relies on an importance sampling distribution to efficiently estimate posterior statistical moments under new target distributions without requiring repeated, computationally prohibitive sampling procedures. Here, the original uniform Sobol sampling space (grey dashed lines) was re-weighted to a posterior likelihood (solid line) by applying a Gaussian prior centered on the nominal parameter values with a 10% standard deviation. **a**, **c**, Probability densities of maximum intracellular $A\beta_{reelin,L}$ concentration in Re+ ECLII and LR neurons and comparison of the two predicted mean values. **b**, **d** Probability densities of maximum $tau_{p,agg}$ concentration in Re+ ECLII (b) and LR (d) neurons and comparison of the two predicted mean values.

4. *Taking this last point further, I find the overall approach of forward simulating a model given parameters and arguing that results are reasonable to be less than modern. In 2026, we know how to perform Bayesian inversion of ODEs given (1) a small number of observed quantities and (2) careful prior specification of probable parameter values. The authors' science would benefit from such an approach, although I would consider such efforts as outside the scope of this manuscript*.

Response: Although we are not asked to do Bayesian inversion of the ODE's, we would be grateful if the reviewer could provide us with some real world examples resembling our case with regard to number of parameters and state variables, lack of any prior information about probability distributions, lack of any time series data on the state variables except for an endpoint range for one state variable, and the presence of an external stochactic driving function (I(t)). We have not been able to find such papers, and before we see them, we are inclined to maintain that, for the type of problem we address in this paper, making inferences about the robustness of predictions by forward simulation with fixed parameter values combined with a global sensitivity analysis is an approach that does not qualify as being less than modern.

Reviewer #2

1. *This study of Kobro-Flatmoen et al., extends a previously published 4-diffrential equation model (Kobro-Flatmoen & Omholt, 2025) into a 7-variable system simulating the decades-long accumulation of Aβ42-reelin complexes (Aβreelin,L) in Re+ECLII entorhinal neurons under recurrent inflammation over a 22-year period (ages 50–72). The central claim is that the particularly high reelin expression in these neurons, combined with senescence-driven inflammation and lysosomal saturation/dysfunction, causes Aβreelin,L complexes tagged for lysosomal degradation to fill up to 80% of soma volume, consistent with seminal observations of early intraneuronal accumulation in AD (D'Andrea et al), while low-reelin (LR) cortical neurons show no such accumulation. The model further links this overload to p-tau fragment production critical for tauopathy, and recapitulates the protective RELN-COLBOS mutation phenotype. The code and data that produce the results and figures have been made available on Zenodo (https://zenodo.org/records/15680979)*

The identification of reelin as a "molecular amplifier" of intraneuronal Aβ accumulation is conceptually Original and well-motivated hypothesis. The framing, ruling out accumulation of pure Aβ42 assemblies by volume calculation and motivating a large binding partner is logically compelling.
The model reproduces 20–80% soma volume occupancy using parameters fixed from the prior published model, which is a notable methodological strength. Reducing the binding affinity γ recapitulates the protective mutation phenotype (suppressed Aβreelin accumulation, maintained GSK3β inhibition, minimal p-tau) in a testable way. Measured rat reelin gradients predict the empirically observed ECLII-before-ECLIII degeneration order. Robustness is demonstrated across simulations with extensive Aβreelin,L accumulation in Re+ECLII neurons and none in LR neurons across parameter space.

Response: We would like to thank the reviewer for his positive evaluation of the paper and for a thorough constructive review. In the following we address all the concerns listed.

2. *Although the study of Kobro-Flatmoen et al., (2023, ref 20) showed bt proximity ligation assays an Aβ/reelin indication, and that the levels of reelin decreased the intraneuronal Aβ load, the entire quantitative design is based on the premise that Aβ42-reelin complexes constitute the soma-filling granules initially observed (D'Andrea et al). No direct immunohistochemical co-localization of reelin with these granules in human AD tissue exists. This must be stated notably in the abstract and conclusions, not only in the discussion.*

Response: We have made revisions in the Abstract, the Introduction, and in the Conclusion that hopefully makes it more clear that the study stands mainly on the premise that reelin functions as a sink for *Aβ.* However, there is in fact evidence for the "*immunohistochemical co-localization of reelin with these granules in human AD tissue*", and this is from own work. Although discussed in the 2023 paper [20], we now realize we should have mentioned this explicitly. Thus, in the Introduction, we have changed the sentence related to the PLA-based co-localization to the following: "in these neurons, Aβ co-localizes with reelin immunohistochemically [19], and it was recently shown that reelin and Aβ42 most likely make a stable complex (Aβ42reelin) [20]". The added reference [19] is Kobro-Flatmoen et al. 2016, doi: 10.1016/j.nbd.2016.05.012.

3. *The 1:1 stoichiometry (Aβ:reelin) is poorly justified. The model assumes one reelin binds one Aβ42. Given reelin's size (~388 kDa), multiple binding sites are plausible. The sensitivity of soma volume occupancy predictions to this assumption is never explored, despite being potentially as influential as any other parameter. In our understanding and by calculation,*

each reelin molecule contributes ~86× the volume of a single Aβ42. The Aβ:reelin complex acts as a volumetric amplifier, making the observed 20–80% soma occupancy quantitatively achievable under the modeled production rates. This is the core quantitative argument for why reelin must be the dominant component of the intraneuronal aggregates, and why the unverified 1:1 stoichiometry assumption is so consequential: if each reelin bound even 2–3 Aβ42 molecules, the volumetric amplification per Aβ42 produced would be halved or thirded, substantially changing the predicted accumulation timescales.

Response: We agree that we should have been more explicit regarding this possibility of another stoichiometry. We have now added a new third paragraph in the Discussion and two new supplementary figures that deal with this issue. We show that a 3:1 stoichiometry does not affect the main conclusions of the study as long as we assume that the immune response times should be similar in the two cases.

4. *GSK3βtot differs 5-fold between LR and Re+ECLII neurons (Table 1) but is not discussed in the main text. Since GSK3β activity drives tau phosphorylation in the model, this difference could independently account for much of the p-tau phenotype, complicating attribution to the Aβ:reelin pathway.*

Response: We specify that the 2025 original model paper [13] should be consulted for justification of the values of GSK3βtot and several other parameters values in the subsection "Parameterization of the differential equations". In that paper we provide a lengthy justification of why the GSK3βtot value are markedly different in LR and Re+ neurons and we would prefer not to repeat it in this paper. However, the main argument is that the two neuron types are supposed to have identical GSK3β regulation (supported by our own experimental data) such that the functional relationships that determine the propagation of the signal between reelin and GSK3β are the same. That is, both groups of neurons produce (or possibly receive in the case of LR neurons) sufficient amounts of reelin to suppress the activation of p-tau formation through GSK3β under native physiological conditions, and this suppression begins to fail when the intracellular level of free reelin falls below a certain threshold. If the Re+ neurons had the same value GSK3βtot, it would mean that the response time would be considerably higher than that of LR neurons in terms of how fast the cell can ramp up its rate of p-tau production associated with an increase in the rate of Aβ production. Since we find this implausible, the $GSK3\beta_{tot}$ value was increased in Re+ neurons to allow a similar response time regarding activation of p-tau production (see last panel in Figs. 2 and 3 in the original model paper). However, this increase does not imply that the p-tau production rate is higher in Re+ neurons compared to LR neurons during a short-term dynamic response to a temporary inflammation event, as shown in the last panel in Figs. S5 and S6. So we do not see how "this difference could independently account for much of the p-tau phenotype, complicating attribution to the Aβ:reelin pathway*".*

5. *The authors acknowledge no direct experimental evidence for the Aβreelin–tau fragment link. Looking at S10 Fig carefully, the parameter that dominates the Sobol indices on the x-axis is ω (omega) in the LR neuron panel where ω shows a first-order index of essentially 1.0. Looking at the Re+ECLII neuron panel of S10 Fig, both ω and θ2 show substantial Sobol indices, with θ2 being the most influential parameter. The p-tau predictions thus depend almost entirely on two parameters - the tau fragment degradation rate (ω) and the lysosomal overload threshold (θ2) - neither of which has experimental support. These predictions must be framed clearly as hypothesis-generating, not quantitatively predictive.*

Response: We are not sure we are able to follow this reasoning. In the discussion we explicitly stated that "Currently, there is no direct experimental evidence for the hypothesized relationship between $A\beta42_{reelin}$ and the production rate of tau fragments." Thus, the hypothesis is that there is such a relationship, and based on this hypothesis we use the

model to test whether it can quantitatively predict a clear difference between the endpoint values of aggregation-prone truncated forms of p-tau in Re+ and LR neurons, which it does. Thus, instead of these predictions being hypothesis-generating, they are hypothesis-supporting. And they are quantitatively predictive conditional on that there is such a relationship and that the two most sensitive parameters are not too far off the mark. That being said, we agree with the reviewer that we should have been more explicit regarding the uncertainties attached to this prediction. Therefore, we have expanded (including the Sobol results pointed out by the reviewer) and revised the second paragraph in the Discussion to stress that before constraining experimental data become available, the quantitative predictions only show that the hypothesized relationship can in principle markedly contribute to enhanced production aggregation-prone truncated forms of p-tau. If we have misunderstood the reviewer's intentions with his comment, we will of course adjust to this.

6. *Equation (7) includes no feedback from [taup,agg] back onto earlier equations, for example on Aβ production. This could be a limitation depending on the intended biological scope. The authors appear to have bounded the model to the intraneuronal immune response rather than the full AD cascade in which this feedback would make sense. This is a defendable modelling choice that should be stated explicitly.*

Response: In the subsection "Robustness of the $A\beta_{reelin,L}$ accumulation results" in the original manuscript, we stated:

"It should be noted that in the model we have not explicitly included any positive feedback effect on $A\beta42$ production rate from increasing levels of $A\beta_{reelin,L}$ and $tau_{p,agg}$. Hence, it is plausible that the number and duration of extracellularly driven inflammation events needed for severe aggregation of $A\beta_{reelin,L}$ are less substantial than the above estimates suggest."

In the revised version, we have deleted this formulation and instead included a new paragraph at the end of the subsection describing Equation (7) that hopefully is considered to be more explicit (and more accurate).

7. *From a biological standpoint, what explains such an intracellular reelin accumulation in a very restricted neuronal population, knowing that reelin is predominantly secreted?*

Response: In a recent purely experimental publication (https://doi.org/10.1007/s00429-024-02816-1) we (AKF, SWO) proposed an explanation for the observed reelin gradient in LII cells that at least to some degree addresses this question:

"We propose that the reason why Re+ECLII neurons display the reelin and Bnip3 gradients is tied to the level of spatial and temporal detail by which they encode information about the external environment. Most of the entorhinal input to the hippocampal formation arises from layers II and III, and the Re+ECLII neurons make up the exclusive layer II population that projects to the dentate gyrus and the CA3/2 hippocampal fields (Varga et al. 2010). In rodents, EC-neurons located close to the rhinal fissure are more strongly connected to the dorsal part of the hippocampal formation, while successively more ventrally situated EC neurons are most strongly connected to correspondingly more ventral levels of the hippocampus (Kobro-Flatmoen and Witter 2019; Canto et al. 2008). Intriguingly, the level of detail by which spatial information about the external environment is encoded is highest at the dorsal extreme of the hippocampus (or the corresponding posterior extreme in humans) and gradually decreases as one moves ventrally (or anteriorly in humans) (Evensmoen et al. 2015; Kjelstrup et al. 2008; Brun et al. 2008). Arguably, the more detailed the encoded environmental representation, the faster it will become outdated. Thus, to keep track of changes in the spatial environment, high-resolution representations are putatively generated and modified by the entorhinal-hippocampal system much more frequently than low-resolution ones. This conception is consistent with data on the only type of entorhinal neuron for which we know the level of detail encoded, namely

the grid cells in MEC, whose firing form a representation of space by tessellating the environment explored by the animal to form a hexagonal grid pattern (Hafting et al. 2005). Thus, grid cells with the most detailed grid pattern sit close to the rhinal fissure, while the grid cells with increasingly less detailed grid patterns are located increasingly farther away from the rhinal fissure (Stensola et al. 2012). It is reasonable to expect that the need to update representations of the environment follows the scale under consideration. For example, several relevant details will change between the different times a rodent traverses a given stretch in its natural habitat, but the distal landmarks remain the same. On the assumption that levels of detail will prove to be encoded along the same gradient also for other, non-spatial representations of the environment, such as those of time or event sequences (Tsao et al. 2018), and objects (Tsao et al. 2013), we anticipate that the graded expression levels of reelin and Bnip3 will turn out to be directly related to the frequency with which it is necessary to update environmental representations in the broad sense. Since reelin is a canonical synaptogenic protein (Qiu et al. 2006; Rogers et al. 2011) heavily involved in the remolding of synaptic contacts (Wasser and Herz 2017), it makes at least perfect sense that its expression is highest in those neurons where the environmental representations are most frequently updated. And since this remolding must necessarily also be energetically demanding, this appears to explain why the Bnip3 expression is so closely tied to the reelin expression in Re+ECLII neurons."

We still think this argument is sensible, but it is surely hypothetical and there may of course be other reasons for why there is so much reelin in ECLII neurons.

8. *No positive feedback from Aβ:reelin to neuroinflammation has been considered. This plausible loop could substantially shorten predicted timescales.*

Response: Please see our response to point 6, which we think covers this concern.

9. *Notation inconsistencies appear in the text (Aβ42reelin / Aβreelin / [Aβreelin,L])*

Response: Yes, we should have been more careful with the notation. We have now made a substantial number of edits that hopefully have removed these inconsistencies. Please advice if we have missed some.
