# Supplementary material for: High reelin expression may explain why a subgroup of entorhinal cortex neurons functions as an initial nucleation site of Alzheimer’s disease
Source: PLoS Comput Biol. 2026 Aug 25;22(8):e1014532. doi: 10.1371/journal.pcbi.1014532 (PMC13506104; doi:10.1371/journal.pcbi.1014532)
Supplement: Peer review history

# Peer review: Decision Letter 0

**DOI:** 10.1371/journal.pcbi.1014532.r001

**Authors:** Andrew Goryachev, Christian Keitel

24 Apr 2026

High reelin expression may explain why a subgroup of entorhinal cortex neurons functions as an initial nucleation site of Alzheimer’s disease

PLOS Computational Biology

Dear Dr. Kobro-Flatmoen,

Thank you for submitting your manuscript to PLOS Computational Biology. After careful consideration, we feel that it has merit but does not fully meet PLOS Computational Biology's publication criteria as it currently stands. Therefore, we invite you to submit a revised version of the manuscript that addresses the points raised during the review process.

Please submit your revised manuscript by Jun 24 2026 11:59PM. If you will need more time than this to complete your revisions, please reply to this message or contact the journal office at ploscompbiol@plos.org. When you're ready to submit your revision, log on to https://www.editorialmanager.com/pcompbiol/ and select the 'Submissions Needing Revision' folder to locate your manuscript file.

Please include the following items when submitting your revised manuscript:

* A letter that responds to each point raised by the editor and reviewer(s). You should upload this letter as a separate file labeled 'Response to Reviewers'. This file does not need to include responses to formatting updates and technical items listed in the 'Journal Requirements' section below.

* A marked-up copy of your manuscript that highlights changes made to the original version. You should upload this as a separate file labeled 'Revised Manuscript with Track Changes'.

* An unmarked version of your revised paper without tracked changes. You should upload this as a separate file labeled 'Manuscript'.

If you would like to make changes to your financial disclosure, competing interests statement, or data availability statement, please make these updates within the submission form at the time of resubmission. Guidelines for resubmitting your figure files are available below the reviewer comments at the end of this letter

We look forward to receiving your revised manuscript.

Kind regards,

Christian Keitel

Academic Editor

PLOS Computational Biology

Andrew Goryachev

Section Editor

PLOS Computational Biology

**Additional Editor Comments:**

Your manuscript has been reviewed by two expert reviewers and both see merit in the work. While Reviewer 1 asks for more context in the light of another publication and has some suggestions for the statistical analysis, Reviewer 2 has more targeted concerns regarding the details of the modelling itself. Please consider these points carefully, with the exception of the last point of Reviewer 1, which I will leave at your discretion.

**Journal Requirements:**

1) Please ensure that the CRediT author contributions listed for every co-author are completed accurately and in full.

At this stage, the following Authors/Authors require contributions: Asgeir Kobro-Flatmoen, Jagir R. Hussan, Peter J Hunter, and Stig W. Omholt. Please ensure that the full contributions of each author are acknowledged in the "Add/Edit/Remove Authors" section of our submission form.

The list of CRediT author contributions may be found here: https://journals.plos.org/ploscompbiol/s/authorship#loc-author-contributions

2) We ask that a manuscript source file is provided at Revision. Please upload your manuscript file as a .doc, .docx, .rtf or .tex. If you are providing a .tex file, please upload it under the item type u2018LaTeX Source Fileu2019 and leave your .pdf version as the item type u2018Manuscriptu2019.

3) Please upload all main figures as separate Figure files in .tif or .eps format. For more information about how to convert and format your figure files please see our guidelines:

https://journals.plos.org/ploscompbiol/s/figures

4) Please amend your detailed Financial Disclosure statement. This is published with the article. It must therefore be completed in full sentences and contain the exact wording you wish to be published.

- State the initials, alongside each funding source, of each author to receive each grant. For example: "This work was supported by the National Institutes of Health (####### to AM; ###### to CJ) and the National Science Foundation (###### to AM)."

- State what role the funders took in the study. If the funders had no role in your study, please state: "The funders had no role in study design, data collection and analysis, decision to publish, or preparation of the manuscript.".

If you did not receive any funding for this study, please simply state: u201cThe authors received no specific funding for this work.u201d

**Reviewers' comments:**

Reviewer's Responses to Questions

**Comments to the Authors:**

**Please note here if the review is uploaded as an attachment.**

Reviewer #1: The authors develop a computational model that uses high levels of reelin in ECLII neurons to explain high levels of AB42 in the same cells that would be otherwise difficult to explain. The importance is that ECLII has been implicated as a birthplace of Alzheimer’s disease (AD) progression, so the mechanistic model advanced potentially captures/explains the earliest of AD cascading dynamics. The model is an ODE model that captures the effect of intermittent, short-term increased production of AB42 and is driven by a random vector of inflammation events increasing production of AB42. The paper is well written and the modeling decisions carefully follow experimental data and previous studies in the AD literature.

On the other hand, the model builds on work recently published in the journal [14]. The specific modeling contribution seems to be the careful extension of a previous model—implicating reelin levels in AB42 growth over a time frame of hours for healthy(?) neurons—to a time frame of 22 years for senescent neurons. It would be helpful if the authors could more clearly delineate the work’s contribution with respect to [14].

While the model parameter specifications are well-articulated, I question the use of fixed parameter values. It would make more sense to specify probability distributions on parameter values. This would provide a better idea of the range of possible outcomes and robustness of the model. I would hope to see this point seriously attended to in a revision.

Taking this last point further, I find the overall approach of forward simulating a model given parameters and arguing that results are reasonable to be less than modern. In 2026, we know how to perform Bayesian inversion of ODEs given (1) a small number of observed quantities and (2) careful prior specification of probable parameter values. The authors’ science would benefit from such an approach, although I would consider such efforts as outside the scope of this manuscript.

Reviewer #2: This study of Kobro-Flatmoen et al., extends a previously published 4-diffrential equation model (Kobro-Flatmoen & Omholt, 2025) into a 7-variable system simulating the decades-long accumulation of Aβ42-reelin complexes (Aβreelin,L) in Re+ECLII entorhinal neurons under recurrent inflammation over a 22-year period (ages 50–72). The central claim is that the particularly high reelin expression in these neurons, combined with senescence-driven inflammation and lysosomal saturation/dysfunction, causes Aβreelin,L complexes tagged for lysosomal degradation to fill up to 80% of soma volume, consistent with seminal observations of early intraneuronal accumulation in AD (D’Andrea et al), while low-reelin (LR) cortical neurons show no such accumulation. The model further links this overload to p-tau fragment production critical for tauopathy, and recapitulates the protective RELN-COLBOS mutation phenotype. The code and data that produce the results and figures have been made available on Zenodo (https://zenodo.org/records/15680979)

Strengths

The identification of reelin as a “molecular amplifier” of intraneuronal Aβ accumulation is conceptually Original and well-motivated hypothesis. The framing, ruling out accumulation of pure Aβ42 assemblies by volume calculation and motivating a large binding partner is logically compelling.

The model reproduces 20–80% soma volume occupancy using parameters fixed from the prior published model, which is a notable methodological strength. Reducing the binding affinity γ recapitulates the protective mutation phenotype (suppressed Aβreelin accumulation, maintained GSK3β inhibition, minimal p-tau) in a testable way. Measured rat reelin gradients predict the empirically observed ECLII-before-ECLIII degeneration order. Robustness is demonstrated across simulations with extensive Aβreelin,L accumulation in Re+ECLII neurons and none in LR neurons across parameter space.

However, some concerns listed below need to be addressed

- Although the study of Kobro-Flatmoen et al., (2023, ref 20) showed bt proximity ligation assays an Aβ/reelin indication, and that the levels of reelin decreased the intraneuronal Aβ load, the entire quantitative design is based on the premise that Aβ42-reelin complexes constitute the soma-filling granules initially observed (D’Andrea et al). No direct immunohistochemical co-localization of reelin with these granules in human AD tissue exists. This must be stated notably in the abstract and conclusions, not only in the discussion.

- the 1:1 stoichiometry (Aβ:reelin) is poorly justified. The model assumes one reelin binds one Aβ42. Given reelin’s size (~388 kDa), multiple binding sites are plausible. The sensitivity of soma volume occupancy predictions to this assumption is never explored, despite being potentially as influential as any other parameter. In our understanding and by calculation, each reelin molecule contributes ~86× the volume of a single Aβ42. The Aβ:reelin complex acts as a volumetric amplifier, making the observed 20–80% soma occupancy quantitatively achievable under the modeled production rates. This is the core quantitative argument for why reelin must be the dominant component of the intraneuronal aggregates, and why the unverified 1:1 stoichiometry assumption is so consequential: if each reelin bound even 2–3 Aβ42 molecules, the volumetric amplification per Aβ42 produced would be halved or thirded, substantially changing the predicted accumulation timescales.

- GSK3βtot differs 5-fold between LR and Re+ECLII neurons (Table 1) but is not discussed in the main text. Since GSK3β activity drives tau phosphorylation in the model, this difference could independently account for much of the p-tau phenotype, complicating attribution to the Aβ:reelin pathway.

- The authors acknowledge no direct experimental evidence for the Aβreelin–tau fragment link. Looking at S10 Fig carefully, the parameter that dominates the Sobol indices on the x-axis is ω (omega) in the LR neuron panel where ω shows a first-order index of essentially 1.0. Looking at the Re+ECLII neuron panel of S10 Fig, both ω and θ2 show substantial Sobol indices, with θ2 being the most influential parameter. The p-tau predictions thus depend almost entirely on two parameters - the tau fragment degradation rate (ω) and the lysosomal overload threshold (θ2) - neither of which has experimental support. These predictions must be framed clearly as hypothesis-generating, not quantitatively predictive.

- Equation (7) includes no feedback from [taup,agg] back onto earlier equations, for example on Aβ production. This could be a limitation depending on the intended biological scope. The authors appear to have bounded the model to the intraneuronal immune response rather than the full AD cascade in which this feedback would make sense. This is a defendable modelling choice that should be stated explicitly.

Additional concerns

- From a biological standpoint, what explains such an intracellular reelin accumulation in a very restricted neuronal population, knowing that reelin is predominantly secreted?

- No positive feedback from Aβ:reelin to neuroinflammation has been considered. This plausible loop could substantially shorten predicted timescales.

- Notation inconsistencies appear in the text (Aβ42reelin / Aβreelin / [Aβreelin,L])

In conclusion, this is an ambitious and mechanistically coherent manuscript that proposes a plausible, original, and computationally supported explanation for one of the core mechanisms in AD biology. The robustness of the Aβ :reelin accumulation result and the RELN-COLBOS recapitulation are real strengths. The work is best understood as a hypothesis-generating computational framework. The framing in the abstract and conclusions should reflect this more clearly. With significant revisions, particularly making the core assumption more explicit, addressing stoichiometry sensitivity, and moderating p-tau claims, this would be a valuable contribution

**********

**Have the authors made all data and (if applicable) computational code underlying the findings in their manuscript fully available?**

The PLOS Data policy requires authors to make all data and code underlying the findings described in their manuscript fully available without restriction, with rare exception (please refer to the Data Availability Statement in the manuscript PDF file). The data and code should be provided as part of the manuscript or its supporting information, or deposited to a public repository. For example, in addition to summary statistics, the data points behind means, medians and variance measures should be available. If there are restrictions on publicly sharing data or code —e.g. participant privacy or use of data from a third party—those must be specified.

Reviewer #1: Yes

Reviewer #2: Yes

**********

PLOS authors have the option to publish the peer review history of their article (what does this mean?). If published, this will include your full peer review and any attached files.

If you choose “no”, your identity will remain anonymous but your review may still be made public.

**Do you want your identity to be public for this peer review?**  For information about this choice, including consent withdrawal, please see our Privacy Policy.

Reviewer #1: No

Reviewer #2: No

[NOTE: If reviewer comments were submitted as an attachment file, they will be attached to this email and accessible via the submission site. Please log into your account, locate the manuscript record, and check for the action link "View Attachments". If this link does not appear, there are no attachment files.]

**Figure resubmission:**

After uploading your figures to PLOS’s NAAS tool - https://ngplosjournals.pagemajik.ai/artanalysis, NAAS will process the files provided and display the results in the "Uploaded Files" section of the page as the processing is complete. If the uploaded figures meet our requirements (or NAAS is able to fix the files to meet our requirements), the figure will be marked as "fixed" above. If NAAS is unable to fix the files, a red "failed" label will appear above. When NAAS has confirmed that the figure files meet our requirements, please download the file via the download option, and include these NAAS processed figure files when submitting your revised manuscript.

**Reproducibility:**

?>

# Author comment: Author response to Decision Letter 1

**DOI:** 10.1371/journal.pcbi.1014532.r002

4 Jun 2026

Attachment Submitted filename: Response to Reviewers.pdf

# Peer review: Decision Letter 1

**DOI:** 10.1371/journal.pcbi.1014532.r003

**Authors:** Andrew Goryachev

3 Jul 2026

Dear Dr. Kobro-Flatmoen,

We are pleased to inform you that your manuscript 'High reelin expression may explain why a subgroup of entorhinal cortex neurons functions as an initial nucleation site of Alzheimer’s disease' has been provisionally accepted for publication in PLOS Computational Biology.

Before your manuscript can be formally accepted you will need to complete some formatting changes, which you will receive in a follow up email. A member of our team will be in touch with a set of requests.

Please note that your manuscript will not be scheduled for publication until you have made the required changes, so a swift response is appreciated.

IMPORTANT: The editorial review process is now complete. PLOS will only permit corrections to spelling, formatting or significant scientific errors from this point onwards. Requests for major changes, or any which affect the scientific understanding of your work, will cause delays to the publication date of your manuscript.

Should you, your institution's press office or the journal office choose to press release your paper, you will automatically be opted out of early publication. We ask that you notify us now if you or your institution is planning to press release the article. All press must be co-ordinated with PLOS.

Thank you again for supporting Open Access publishing; we are looking forward to publishing your work in PLOS Computational Biology.

Best regards,

Andrew Goryachev

Section Editor

PLOS Computational Biology

***********************************************************

Reviewer's Responses to Questions

**Comments to the Authors:**

**Please note here if the review is uploaded as an attachment.**

Reviewer #1: I am satisfied with the authors' response. I also insist that their questions regarding Bayesian inversion for this specific modeling context point to potential substantive methodological contributions to the field.

Reviewer #2: The authors have responded substantially to most concerns raised in the first review. The manuscript has improved meaningfully and the revision is satisfactory.

**********

**Have the authors made all data and (if applicable) computational code underlying the findings in their manuscript fully available?**

The PLOS Data policy requires authors to make all data and code underlying the findings described in their manuscript fully available without restriction, with rare exception (please refer to the Data Availability Statement in the manuscript PDF file). The data and code should be provided as part of the manuscript or its supporting information, or deposited to a public repository. For example, in addition to summary statistics, the data points behind means, medians and variance measures should be available. If there are restrictions on publicly sharing data or code —e.g. participant privacy or use of data from a third party—those must be specified.

Reviewer #1: Yes

Reviewer #2: Yes

**********

PLOS authors have the option to publish the peer review history of their article (what does this mean?). If published, this will include your full peer review and any attached files.

If you choose “no”, your identity will remain anonymous but your review may still be made public.

**Do you want your identity to be public for this peer review?**  For information about this choice, including consent withdrawal, please see our Privacy Policy.

Reviewer #1: No

Reviewer #2: No

# Editor report: Acceptance letter

**DOI:** 10.1371/journal.pcbi.1014532.r004

**Authors:** Andrew Goryachev

PCOMPBIOL-D-26-00426R1

High reelin expression may explain why a subgroup of entorhinal cortex neurons functions as an initial nucleation site of Alzheimer’s disease

Dear Dr Kobro-Flatmoen,

I am pleased to inform you that your manuscript has been formally accepted for publication in PLOS Computational Biology. Your manuscript is now with our production department and you will be notified of the publication date in due course.

The corresponding author will soon be receiving a typeset proof for review, to ensure errors have not been introduced during production. Please review the PDF proof of your manuscript carefully, as this is the last chance to correct any errors. Please note that major changes, or those which affect the scientific understanding of the work, will likely cause delays to the publication date of your manuscript.

Soon after your final files are uploaded, unless you have opted out, the early version of your manuscript will be published online. The date of the early version will be your article's publication date. The final article will be published to the same URL, and all versions of the paper will be accessible to readers.

For Research, Software, and Methods articles, you will receive an invoice from PLOS for your publication fee after your manuscript has reached the completed accept phase. If you receive an email requesting payment before acceptance or for any other service, this may be a phishing scheme. Learn how to identify phishing emails and protect your accounts at https://explore.plos.org/phishing.

Thank you again for supporting PLOS Computational Biology and open-access publishing. We are looking forward to publishing your work!

With kind regards,

Sharmila Kamatchi

PLOS Computational Biology | Carlyle House, Carlyle Road, Cambridge CB4 3DN | United Kingdom ploscompbiol@plos.org | Phone +44 (0) 1223-442824 | ploscompbiol.org | @PLOSCompBiol
